# Supplementary material for: Locally-curved geometry generates bending cracks in the African elephant skin
Source: Nat Commun. 2018 Oct 2;9:3865. doi: 10.1038/s41467-018-06257-3 (PMC6168576; doi:10.1038/s41467-018-06257-3)
Supplement: Supplementary file 1 — Supplementary Information [file 41467_2018_6257_MOESM1_ESM.pdf]

Supplementary Information for

Locally-Curved Geometry Generate Bending Cracks in The African Elephant Skin

António F. Martins[1,2], Nigel C. Bennett[3], Sylvie Clavel[4], Herman Groenewald[5], Sean Hensman[6], Stefan Hoby[7], Antoine Joris[8], Paul R. Manger[9], and Michel C. Milinkovitch[1,2,*]

correspondence to: Michel.Milinkovitch@unige.ch

This PDF file includes:

Supplementary Methods
Supplementary Discussion
Supplementary Figs. 1 to 20
Supplementary Tables 1 to 4

Supplementary Methods

Numerical model

Energy contributions. The simulations were performed using a damped Newtonian dynamics framework in which the nodal forces (excluding damping) were derived from the total energy, E, via the usual relation $\boldsymbol{F} = -\nabla E$ (see *'Implementation details'* below). We considered five different contributions to the system's energy: spring, volumetric, bending, substrate and contact energies. The first two of these correspond to the stretching response of the shell and are needed to simulate materials with a Poisson's ratio larger than 1/3 (see below).

(I) Spring energy. An edge connecting the ith and j^{th} nodes of the mesh is modeled as a spring s contributing with an energy

$$E_s^{Spring} = \frac{1}{2} K_s \left(\| \boldsymbol{r}_i - \boldsymbol{r}_j \| - l_s^0 \right)^2, \tag{1}$$

where K_s and l_s^0 are the spring's stiffness and rest length, respectively. In addition, $K_s = K_s^{sh} + K_s^{sub,\|}$, *i.e.*, K_s includes contributions from both the shell's (K_s^{sh}) and the substrate's ($K_s^{sub,\|}$) elastic response (Supplementary Fig. 7). The total spring energy is thus

$$E^{Spring} = \sum_{s \in S} E_s^{Spring}, \tag{2}$$

where S is the set of all springs/edges.

(II) Volumetric energy. It is well-known that models employing solely 'central force' springs can only simulate the stretching response of materials with $\nu = 1/3$ (under 2D plane stress conditions) (*1*). Since the material we want to simulate (partially dry *stratum corneum*) has a Poisson's ratio of ≈0.40, we implemented a slightly modified version of the volume-compensated method recently proposed by (*2*), in which local volumetric terms are added to the system's energy. This scheme has been shown to perform well in elasticity, plasticity and fracture studies (*2-4*). For a given node n, the volumetric energy contribution takes the form

$$E_n^{Vol} = \frac{1}{4} K_n^{vol} \left(\sum_{m \in Adj(n)} \| \boldsymbol{r}_n - \boldsymbol{r}_m \| - l_{s_{nm}}^0 \right)^2, \tag{3}$$

where K_n^{vol} is the node's volumetric constant (related to the material's elastic moduli, see the *'Supplementary Discussion'* section), *Adj(n)* is the set of nodes adjacent to node n, and s_{nm} is the spring connecting nodes n and m. The total volumetric energy reads

$$E^{Vol} = \sum_{n \in N} E_n^{Vol}, \tag{4}$$

where N is the set of all nodes.

That this combination of spring and volumetric energies can be used to model the stretching response of any linear material can be intuitively understood by comparing equations (1) expressed in nodal form (which yields a term proportional to $\sum \left(l_{ij} - l_{ij}^0 \right)^2$) and (3) ($\propto \left(\sum l_{ij} - l_{ij}^0 \right)^2$) with the strain-energy density function of a Saint Venant-

Kirchhoff material, $W(\boldsymbol{E}) = \mu tr(\boldsymbol{E}^2) + \frac{\lambda}{2}(tr\boldsymbol{E})^2$ (here, $\boldsymbol{E}$ stands for the Green-Lagrange strain tensor and not the energy). It is readily evident that there is a parallel between the energy contributions of the two formulations.

(III) Bending energy. To model the bending response of the shell, we extended the method originally proposed by (*5, 6*) for the study of plates (shells with a flat rest configuration). The contribution of two adjacent faces α and β to the bending energy is

$$E_{\langle\alpha,\beta\rangle}^{Bending} = \frac{1}{2} K_{\langle\alpha,\beta\rangle}^{bnd} \|\Delta\boldsymbol{n} - \Delta\boldsymbol{n}^0\|^2, \tag{5}$$

with $\Delta\boldsymbol{n} = \boldsymbol{n}_\alpha - \boldsymbol{n}_\beta$ and $\Delta\boldsymbol{n}^0 = \boldsymbol{n}_\alpha^0 - \boldsymbol{n}_\beta^0$. Here, $K_{<\alpha,\beta>}^{bnd}$ is the pair's bending constant, while $\boldsymbol{n}_{\alpha,\beta}$ and $\boldsymbol{n}^0_{\alpha,\beta}$ are the faces' current and non-deformed orientations, respectively. Equation (5) follows from the discretization of the bending term of the continuous Koiter shell model (*7*). Note that for a flat rest configuration one has $\boldsymbol{n}^0_\alpha = \boldsymbol{n}^0_\beta \rightarrow \Delta\boldsymbol{n}^0 = \boldsymbol{0}$, showing that equation (5) is consistent with the plate model introduced in (*5*). The total bending energy is thus simply given by

$$E^{Bending} = \sum_{\langle\alpha,\beta\rangle\in FP} E_{\langle\alpha,\beta\rangle}^{Bending}, \tag{6}$$

where *FP* is the set of adjacent face pairs.

Since we are not aware of any other publication modeling the bending response of a shell in this manner, we tested our formulation by comparing our numerical results with *(i)* the analytical solution to the problem of unwrapping a cylindrical shell without stretching it, and *(ii)* the recent numerical and experimental study on spherical thin shells subject to contractive substrate forces (*8*). In the first case, the simulated numerical bending energy was always within 0.3% of the theoretically calculated value (we tested cylinders of different radii with partial and total unwrapping), while for the second scenario we were able to reproduce the observed transition from quasi-hexagonal to labyrinth phases, including the intermediate bistable pattern. These results, coupled with the theoretical reasoning behind the derivation of equation (5), support our model as a valid approach to the simulation of bending effects in shells.

(IV) Substrate energy. In our model, the substrate's influence is separated into in-plane and out-of-plane contributions (Supplementary Fig. 7). The former is added through the spring's constant value, K_s (equation (1)), and plays an important role in correctly modeling the behavior of the system when the shell layer starts cracking. The out-of-plane component, on the other hand, is modeled on a per-node basis and, for a node *n*, is given by

$$E_n^{\mathrm{Sub}} = \frac{1}{2} A_n K_n^{\mathrm{sub},\perp} \left(d_n^{\mathrm{sub}}\right)^2, \tag{7}$$

where $K_n^{sub,\perp}$ is the out-of-plane substrate constant, A_n is the nodal area (defined here as the average of the areas of the faces adjacent to node *n*), and d_n^{sub} is the distance between the node and its *current* rest position (*i.e.*, directly 'on top' of the substrate, if the latter is in a stress-free configuration). Note that since the substrate grows during the simulations, the rest position changes accordingly. The total out-of-plane substrate contribution is then

$$E^{\mathrm{Sub}} = \sum_{n\in N} E_n^{\mathrm{Sub}}. \quad (8)$$

(V) Contact energy. Due to the growth and intricate geometry of the substrate, it is possible that the shell may come into contact with itself during the course of a simulation (usually in the trough between two papillae). We model this interaction as a node-face contact potential with a limited range (*9-11*). Concretely, for a node n, the energy contribution is

$$E_n^{Contact} = \sum_{f\in F} E_{n,f}^{Contact}, \quad (9)$$

where F is the set of all faces and

$$E_{n,f}^{Contact} = \begin{cases} \frac{1}{2} A_n K_n^{con} \left(d_C - d_{n,f}\right)^2, & d_{n,f} \leqslant d_C \wedge \boldsymbol{n}_n \cdot \boldsymbol{n}_f < 0 \\ 0 & otherwise \end{cases}, \quad (10)$$

where $d_{n,f}$ is the distance between node n and face f, d_C is the minimal interaction distance, $\boldsymbol{n}_n$ and $\boldsymbol{n}_f$ are the node's and the face's normals respectively, and K_n^{con} is a constant which modulates the strength of the repulsion. The total contact energy is thus

$$E^{Contact} = \sum_{n\in N} E_n^{Contact}. \quad (11)$$

Relation between the model and physical parameters. Connecting the parameters of our custom mass-spring model with physically measurable quantities is not trivial for an arbitrary mesh structure and geometry. However, if we assume that the nodes are approximately organized in a regular hexagonal packing (as is the case in our simulation, see *'Implementation details'* section below), it is possible to establish a number of results relating the two sets of numerical and physical parameters (*1, 2, 6*). Moreover, even when rigorous results are not available, it is often possible to obtain scaling laws based solely on physical arguments (*12-14*). Considering first the stretching response of the shell, which includes both the spring (equation 1) and the volumetric (equation 3) contributions introduced above, it is possible to show that, for a sheet of material with uniform thickness h and subject to plane stress conditions, the following relations hold:

$$K_s^{sh} = \frac{2}{\sqrt{3}(1+\nu)} Yh, \quad (12)$$

$$K_n^{vol} = \frac{3\nu - 1}{6\sqrt{3}(1-\nu^2)} Yh, \quad (13)$$

where Y and ν are the Young's modulus and Poisson's ratio of the shell's material. Additionally, the bending constant (equation 5) can be determined via

$$K_{\langle\alpha,\beta\rangle}^{bnd} = \frac{Yh^3}{6\sqrt{3}(1-\nu^2)}. \quad (14)$$

Exact relations like the ones above are not available for the remaining 'stiffness' constants ($K_s^{sub,\parallel}$, $K_s^{sub,\perp}$ and K_n^{con}). Nevertheless, it is possible to establish a number of simple scaling laws that can be used to derive order of magnitude estimates for these parameters. For $K_s^{sub,\parallel}$ and $K_s^{sub,\perp}$, we borrow results from the physics of wrinkling and folding in the deep substrate limit (*12, 13*). These establish that the substrate deformation has a characteristic length scale, $l_d \sim h(Y/Y_s)^{1/3}$ (*15*), whose value is connected to the stiffness constants via

$$K_n^{\mathrm{sub},\perp} \sim \frac{Y_s}{l_d} \sim \frac{Y}{h}\left(\frac{Y_s}{Y}\right)^{4/3}, \tag{15}$$

$$K_n^{\mathrm{sub},\parallel} \sim Y_s l_d \sim Yh\left(\frac{Y_s}{Y}\right)^{2/3}, \tag{16}$$

where Y_s is the substrate's Young's modulus. In a similar fashion, the value of K_n^{con} can be estimated by assuming that the contact is non-adhesive and Hertzian (*i.e.*, non-frictional), from which the relation $K_n^{con} \sim Y$ follows (*14*). Moreover, it can be shown that consistency with linear elasticity implies that $K_n^{con} \sim 1/h$. It follows, thus, that

$$K_n^{con} \sim \frac{Y}{h}. \tag{17}$$

Finally, by noting that our mesh simulates the shell's mid-surface, the minimal interaction distance for the contact term is simply $d_C \approx h$.

Since we take equations (15-17) to be exact (*i.e.*, we assume that the proportionality constant is ≈1), the results above show that the 7 parameters of our model can be recovered from only 4 physical constants: the dry *stratum corneum*'s Young's modulus Y (which can be set to 1 in our simulations) and Poisson's ratio ν, the ratio Y_s/Y between the Young's moduli of the underlying layers of the skin and of the outermost dry *stratum corneum*, and the dry *stratum corneum* layer's thickness h. To these, we must add a fifth physical quantity: the dry *stratum corneum*'s elongation at break, ε_C.

With the exception of h, estimating these values for the African bush elephant's skin is challenging. Nevertheless, given the similarities between the human and the elephant's skin structures evidenced by our histological analyses (Fig. 3 and Supplementary Fig. 5), we assumed that the values for these physical constants are similar for both species. This allowed us to estimate $\nu \approx 0.40$ (*16*) and $Y_s/Y \approx 0.005$-0.010 (*17*). Fixing ε_C has proven more difficult due to the wide range of values cited in the literature: from 0.02 to 1.00 (*18*). Moreover, it is known that this figure varies drastically with temperature and relative humidity (*19*). Therefore, we performed simulations with different values of ε_C in order to probe the influence of this parameter on the final cracking pattern and statistics. Finally, note that direct thickness measures of the full *stratum corneum* (~200-300μm, in most cases) are overestimates of h, as our shell represents only the outermost, (partially) dry, sheets of that skin layer. Indeed, the *in vivo* water content profile within the *stratum corneum* usually exhibits an approximately sigmoidal shape (*20-22*) which can be broadly divided in three parts:

i. The outermost *stratum corneum* sheets, extending from the skin's surface to about 10-20% of the full layer's thickness. The water content has a relatively flat profile in this

region, and its value is at a minimum. This is the object of our shell simulations, and the parameter h should be taken as the thickness of this part of the *stratum corneum* alone.

ii. The region (i) above is followed by one of rapid increase in water content, typically extending until 50-70% of the full layer's thickness. We expect the *stratum corneum* sheets in this range to be much more hydrated than the ones in the outermost region, and thus have a much lower Young's modulus and much higher elongation at break value (*18*).

iii. A final, innermost region in which the water content profile is both flat and at a maximum. The *stratum corneum* sheets are probably fully hydrated in this part.

In effect, we treat the two inner regions of the *stratum corneum* ((ii) and (iii) above) as part of the substrate in our simulations, given that we expect their mechanical characteristics to be similar to those of the remaining skin layers (viable epidermis and dermis). We can thus conclude that $h \sim$ 20-40µm.

Implementations details. We simulated the substrate (inner *stratum corneum*) growth process (Supplementary Movie 5) in a quasi-static fashion by iterating between the following two steps. First, we perturb the system by displacing the nodal rest positions with respect to the substrate (used in equation (7)) by an amount $\delta\boldsymbol{n}^0$, where $\boldsymbol{n}^0$ is the original orientation of the node and δ is a small quantity (typically, $\delta << h$, where h is the shell's thickness). Second, we evolve the system to the steady state using linearly damped Newtonian dynamics (see below). During this step, we continuously monitor the total strain (stretching and bending) on each edge, breaking it in case this value exceeds a given critical strain (see below). The quasi-static assumption is justified because the process of adding new keratin sheets to the *stratum corneum* (represented in our model by the growth of the substrate) takes place on a timescale of days to weeks, *i.e.*, much longer than that of elastic relaxation.

We let the simulation run for at least N_c cycles, where N_c is chosen such that $N_c\delta \sim$ 300-350µm in physical units (note that $N_c\delta$ is roughly the final thickness of the *stratum corneum* layer if it had grown unimpeded), except for the simulations with ε_c=0.1, as in those cases the system is usually thoroughly cracked by $N_c\delta \sim$ 150-200µm. Since the values we measured for the animal's *stratum corneum* thickness rarely exceed 300µm (especially for young individuals), we assume that at this point the outermost part of this layer is either shed or abraded. In practice, $N_c \sim$ 100-200. Note that in each cycle, the system is converged to the steady state, a step that usually takes 200,000 to 300,000 iterations of the solver. Thus, a complete simulation typically takes between 20 and 60 million iterations. For reasonably large meshes (300k nodes and 600k faces) containing ~50-60 papillae, the running time on a 16-core machine is 5 to 15 days per simulation.

Damped Newtonian dynamics. Convergence to the steady state is attained dynamically using a linearly damped leapfrog algorithm, a standard choice for the numerical integration of classical equations of motion. At each step, the energy-derived nodal forces are first calculated via

$$\boldsymbol{F}_i = -\nabla E_i = -\nabla\left(E_i^{Spring} + E_i^{Vol} + E_i^{Bending} + E_i^{\text{Sub}} + E_i^{Contact}\right). \quad (18)$$

To these, a linear damping force is added ($\boldsymbol{F}_d = -\gamma\boldsymbol{v}$), resulting in the following second-order integration scheme

$$\boldsymbol{v}_{i+1/2} = \frac{(1-\gamma\Delta t/2m)\boldsymbol{v}_{i-1/2}+\Delta t\boldsymbol{F}_i/m}{1+\gamma\Delta t/2m}, \tag{19}$$

$$\boldsymbol{r}_{i+1} = \boldsymbol{r}_i + \Delta t\boldsymbol{v}_{i+1/2}, \tag{20}$$

where $\gamma \sim m$ is the damping parameter, m is the nodal mass (proportional to the nodal area) and $\Delta t \sim \langle L\rangle(h/K_s)^{1/2}$ is the integration step ($\langle L\rangle$ is the mean edge length). We assume that the steady state has been reached if either *(i)* $\frac{max_{n\in N}\|\boldsymbol{r}_{i+1}-\boldsymbol{r}_i\|}{\langle L\rangle} < 10^{-4}$ for 1000 consecutive iterations, or *(ii)* the system's energy shows signs of displaying a cyclic behavior. This second convergence criterion is monitored as follows: if the number of iterations exceeds 50,000, we start probing the total energy at regular intervals (typically every 500 iterations, E^*_i). When a number n^* of these values has been collected (usually n^*=21, such that we have a sequence E^*_0,E^*_1,...,E^*_{20}), we check if (a) $E^*_{k+1} - E^*_k > 0$ at least 35% of the times (*i.e.*, if the energy has regularly increased), (b) $E^*_{20} - E^*_0 > 0$ (*i.e.*, if the energy has increased over a large number of iterations), and (c) no bond was broken during this interval. If these three conditions are met, we assume that the system has reached the steady state. The second convergence criterion is necessary due to the approximate way in which the contact forces are calculated, as discussed in detail below. We have empirically confirmed that it can help the solver escape undesired repetitive behavior without affecting its ability to find the steady state.

Bond breaking. In our model, bond breaking occurs between solver iterations. We calculate the stretching strain on each spring s using

$$\varepsilon_s = \frac{l_s - l_s^0}{l_s^0}, \tag{21}$$

as well as the bending strain for each face pair $\langle\alpha,\beta\rangle$ via

$$\varepsilon_b = \frac{h}{2l^0_{\langle\alpha,\beta\rangle}}\|\Delta\boldsymbol{n} - \Delta\boldsymbol{n}^0\|, \tag{22}$$

where $l^0_{\langle\alpha,\beta\rangle}$ is the rest distance between the faces' centroids. Although the bending strain is positive on one side of the shell and negative on the other, we are only interested in knowing whether the tensile strain is enough to break a bond, hence, we consider only the side where the positive values can be found (*i.e.*, $\varepsilon_b \geq 0$.). However, for a given internal edge, the stretching and bending strains calculated in this way are not aligned. Concretely, the stretching strain given by equation (21) is along the spring's direction, while equation (22) yields a bending strain roughly in the direction of the vector connecting the faces' centroids (*i.e.*, essentially orthogonal to the spring's strain). We correct for this misalignment using a simple fitting procedure: for each edge, we consider the bending strains calculated in its neighborhood and fit a 2D strain tensor to these values. This can then be used to straightforwardly calculate the bending strain in the direction of the edge, ε^e_b. Finally, the total strain for an internal edge is

$$\varepsilon = \varepsilon_s + \varepsilon^e_b, \tag{23}$$

and the breaking condition is

$$\varepsilon > \varepsilon_c. \tag{24}$$

If an edge satisfies this condition, we irreversibly set $K_s^{sh} = 0$ and $K_{<\alpha,\beta>}^{bnd} = 0$ for the associated spring and face pair, respectively. Note, however, that the out-of-plane springs and the edge's substrate spring are left intact. Moreover, we remove the faces from the set F and exclude the contribution of the spring to the volumetric energy (equation 3). For boundary edges the procedure is the same except that only ε_s is considered for the total strain (equation 23).

Contact forces. Given the limited range of the contact energy (equation 10), it is clear that, for reasonable values of d_C (*i.e.*, of the order of the shell's thickness), only very few node-face pairs need to be considered. Nevertheless, since *(i)* it is not possible to determine *a priori* which nodes and faces will interact, and *(ii)* calculating node-to-face distances ($d_{n,f}$) exactly requires a fair amount of algebra, evaluating the contact forces derived from $E^{Contact}$ at each iteration quickly becomes prohibitively time-consuming as the mesh size increases. We addressed these issues by updating the list of interacting node-face pairs only at specific iterations, and by calculating $d_{n,f}$ in an approximate manner. Concretely, we used the following algorithm to calculate the contact forces in our model. At the start of each iteration, we determine whether the list of interacting node-face pairs needs to be updated. This is the case if: *(i)* it is the first iteration, or *(ii)* the list has not been updated for N_{ud} iterations ($N_{ud} \sim 500$), or *(iii)* the cumulative maximum position deviation (*i.e.*, $\sum max_{n\in N} \|\boldsymbol{r}_{i+1} - \boldsymbol{r}_i\|$) since the last update exceeds a certain threshold (typically, $0.1d_C$). If any of the above criteria is met, the centroids of every face in the set F are recalculated and listed. A k-d tree nearest neighbor search is then performed for each node to determine the face centroids that are within a $1.5d_C$ radius of the node's position. For each centroid found, an interacting node-face pair is created provided $\boldsymbol{n}_n \cdot \boldsymbol{n}_f < 0$. In addition, at each iteration, the node-to-face distance $d_{n,f}$ of each interacting node-face pair (needed when evaluating the nodal forces in equation 18) is approximately calculated by discretizing the face in 7 points (its centroid, the vertices and the edges' midpoints), computing the distances between the node and these points and taking $d_{n,f}$ to be the smallest of these distances. The contact force on the node, $\boldsymbol{f}_n$, is then calculated via equations (10) and (18), while the force on the face's vertices is given by $\boldsymbol{f}_i = -w_i\boldsymbol{f}_n$, where w_i are such that *(i)* $w_i \sim 1/(d_{n,fi})^2$, with $d_{n,fi}$ the distance between the node and the face's vertex i, and *(ii)* $\sum_i w_i = 1$.

We have confirmed that the results obtained using this approximated calculation of contact forces are essentially identical to the ones obtained when these terms are computed exactly. The use of such approximations sometimes lead to the solver exhibiting a small amplitude cyclic behavior (*e.g.*, when the distance between a node and two of the face's discretization points is almost the same, the node's position may oscillate with an amplitude $\sim 10^{-2}$-10^{-3} $\langle L \rangle$). While the influence of this behavior on the steady state positions is minor, in practice it may make it impossible for the solver to satisfy traditional convergence criteria based on nodal displacements or velocities. Hence, we devised the alternative convergence criterion explained in the *'Damped Newtonian dynamics'* section above, making the approximation efficient and resulting in a speed-up of 100 to 200 times over the exact calculations.

Mesh geometry and regularity. We obtained the shell's geometry (*i.e.*, its rest configuration) from micro-CT scans of African elephant skin samples from which the *stratum corneum* had been previously removed. However, the raw surface extracted from the CT data is rather rough, exhibiting multiple kinks and a very heterogeneous edge length distribution (Supplementary Figs. 6A,D). As such, it is ill-suited for our simulations due to the approximate regular hexagonal packing assumption used to derive some of the relations between the numerical and physical constants in our model (see above). To address this issue, we used a custom mesh regularization algorithm to smooth out both the fine-scale roughness and the edge length distribution (Supplementary Figs. 6B,D). Note, however, that the large-scale mesh features and geometry are unaffected by this procedure, *i.e.*, the smoothing is only local. The resulting edge length distribution is considerably more regular, with approximately 90% of the edge lengths falling within the interval $0.75 \leq L/\langle L\rangle \leq 1.25$, where $\langle L\rangle$ is the mean edge length. In addition, the resulting node connectivity distribution is strongly peaked around 6: the percentage of nodes with this number of neighbors varies between 70 and 95% (Supplementary Fig. 6C). These two statistics indicate that the node organization of the smoothed meshes approaches that of a regular hexagonal packing, and thus we can expect equations (12-14) to hold approximately.

Stress distribution and crack localization and orientation. As the growth of the substrate (inner *stratum corneum*) progresses, one expects the unshed outer sheets to develop both stretching and bending strains. Our model suggests that the distribution of these is highly heterogeneous (Supplementary Fig. 9A,B): while tensile stretching strains develop mostly over the papillae, with only modestly compressive strains present in the troughs, the bending strain is essentially confined to the troughs. Combining the stretching and bending strains (Supplementary Fig. 9C) leads to a full strain distribution (prior to the onset of cracking) that *(i)* is predominantly tensile, *(ii)* attains its highest (most tensile) values in the trough regions along the direction perpendicular to the trough orientation, and *(iii)* exhibits a marked heterogeneity at trough junctions (see details below). Moreover, we observed these qualitative characteristics for all the values of h (outer *stratum corneum* thickness) that we tested.

The simulated strain distribution sheds some light on the striking observation that cracks appear mostly in the troughs. Indeed, our simulations suggest that bending strains play a key role in confining the cracks to the troughs: not only do they ensure that the cracks appear predominantly in these regions (since that is where the highest strains can be found), but also that the propagation will occur mostly along the direction of the trough (because the full strain is essentially perpendicular to it). Furthermore, our observations of cracked regions using light and electron microscopy (see *'Evidence for cracking as the generative mechanism of the African elephant skin channels'* in the Supplementary Discussion below) revealed a sharp fracture with little or no recoil, consistent with the bending-dominated cracking process we suggest here (Fig. 5). This hypothesis is likewise consistent with the steady pace at which cracks form (Supplementary Fig. 11A and Supplementary Movie 5). Physically, the process is similar to the fracture of a rectangular thin sheet of dry gelatin when it is bent to make two opposing edges meet: although little stretching is present, the strong bending strains (and stresses) in the middle of the sheet can lead to material failure and the formation of a crack in the direction parallel to the edges. To confirm that this strain distribution can indeed confine cracks to the troughs, we used a

simple quantitative metric: the fraction of broken edges (cracks) that appear in the troughs after the onset of cracking (Supplementary Fig. 11B). We found that, in most cases, over 80% of the broken edges appear in trough regions, despite the fact that only ~23% of the edges are located in the troughs. In broad terms, the higher the critical strain, ε_c, the higher the probability that cracks will be found in the troughs for a given growth value. The influence of the layer's thickness, h, is somewhat less pronounced, although it seems that larger values allow simulations to hold the cracks in the troughs for longer times (*i.e.*, until larger values of growth are reached). Superficially, this suggests that our model fits the observed skin pattern better for higher values of ε_c. Our simulations indicate that for $\varepsilon_c \geq 0.20$, the cracks fall almost entirely within the trough region, a result that is consistent with what we observe on the skin of the animal (cracks rarely appear outside the troughs; Fig. 4E). This consistency holds independently of the value of h used in the simulation ($h \approx [20,40]\mu m$).

The strain distribution (Supplementary Fig. 9) can similarly be used to clarify the reasons for the discrepancy between crack and trough angles (see *'Angular Analysis'* section in the Supplementary Discussion below). Indeed, our simulations indicate that the strain distribution at trough junctions is very heterogeneous, varying both from junction to junction and within each junction. Moreover, and although it is by no means a universal feature, we have noticed that in several cases the strain distribution at junctions exhibits a strain-free central region surrounded by a curved triangle where higher tensile strains develop (inset in Supplementary Fig. 9C). These numerical simulation results help explain why *(i)* the angular statistics for cracks and troughs in the animal differ considerably (possibly a byproduct of the variety of strain profiles at junctions), *(ii)* crack junctions have a much larger angular spreading than trough junctions (also a consequence of the mentioned heterogeneity), and *(iii)* a substantial proportion of crack junctions fall within the '90°-135°-135°' profile region (expected if the cracks propagate along the sides of the 'curved triangle' of high strain and meet at one of its vertices).

Additional remarks. Here, we comment on the validity of the approximations used in our physical model and on its potential shortcomings.

- *Thin shell approximation (Kirchhoff shell)*: while the simulated layer's maximum thickness (≈40-50μm) is always much smaller than the in-plane dimensions of the system (and thus the shell is 'thin' in that sense), the principal radii of curvature of the undeformed and deformed shells can locally reach values of ≈80-100μm, *i.e.*, only twice the shell's maximum thickness. Although nonlinear effects due to local variations in shell thickness or through-the-thickness shear are unaccounted for in our model, the contributions of these effects are expected to be small (compared to the dominant terms of the thin shell approximation) because the radii of curvature are still larger than the shell's thickness.
- *Linear elasticity and brittle fracture*: even though the *stratum corneum* exhibits a viscoelastic behavior and a non-linear stress-strain curve when fully hydrated (like many other biological tissues), its mechanical response has been shown to be remarkably linear at lower levels of hydration, with no apparent plastic deformation taking place before fracture, *i.e.*, it fails in a brittle fashion (*17*). Since the outer sheets of the *stratum corneum* in the African elephant are likely poorly hydrated (see above), we expect the use of a linear elastic model with a strain-based failure criterion to be well-justified.

- *Thickness's dependence on growth*: in our simulations, we make the approximation that the thickness (h) of the outermost part of the *stratum corneum* (the 'shell' of our model) does not change during the growth/proliferation of the underlying sheets (the growth of the 'substrate' in the model). This assumption is based on the idea that the thickness of the driest, outermost parts of the *stratum corneum* will be mainly influenced by the external, environmental conditions (such as temperature and relative humidity) rather than by the 'internal' skin conditions. Nevertheless, since we do not expect this assumption to be strictly met, we carried out a number of simulations in which the shell's thickness was assumed to be a function of growth, $h(g)$, and found their results to be very similar (at least qualitatively) to those of simulations with a fixed value of h. Thus, we expect the approximation of h being constant as a reasonable one.
- *Intact stratum corneum patches containing several papillae*: although cracks tend to individualize all papillae (Supplementary Fig. 20A), we observed that, in some regions of the skin, the papillae can sometimes become clumped together, *i.e.*, the *stratum corneum* in between them remains intact, forming irregularly shaped cracked 'patches' containing a small number of papillae (Supplementary Fig. 20B,C). This seems especially likely to happen on the forehead of the animal (Supplementary Fig. 20D). Currently, we lack a comprehensive understanding of how these structures form. Nevertheless, the limited data allows us to speculate on a number of possible influencing factors.
  - ✓ Thickness of simulated layer (outer *stratum corneum*): our preliminary results indicate that thicker shells allow adjacent troughs to escape cracking in some cases, leading to the formation of patches containing several papillae (Supplementary Fig. 20E,F). Superficially, this could fit with the clumping statistics found for different body parts (Supplementary Fig. 20D), as one might expect the *stratum corneum* to be thicker on the forehead and side of the animal relative to its ear (its role as a 'cooling organ' might require a thinner *stratum corneum* for maximum efficiency) and proboscis (likewise, its sensory function may benefit from thinner layers). However, we do not have access to the necessary skin samples systematically collected from a large number of body regions of a single individual elephant to verify this supposition. In addition, simulations with larger thickness values might violate the thin shell approximation of our model, and thus produce possibly unreliable results.
  - ✓ Substrate effects: it is possible that spatially heterogeneous physical properties of the underlying tissue layers (which enter the model via the ratio Y_s/Y) influence the cracking process and lead to the clumping of papillae in some body regions. We do not have access to the necessary data to test that possibility.
  - ✓ *Stratum corneum* regeneration: since new layers of *stratum corneum* are constantly being added to the skin of the animal, cracks can reorganize, 'heal' and widen (Supplementary Figs. 1 and 2). It is thus possible that the continuous regeneration and cracking of the *stratum corneum*, which are not simulated by our model, play a role in clumping the papillae. Nevertheless, given the different degrees of clumping found in different body parts, this factor is unlikely to be solely responsible for the formation of these structures.

- ✓ Other factors: spatial heterogeneities in trough spacing, skin movement or *stratum corneum* physical properties, may likewise contribute to different degrees of clumping in different body areas.

- *Arrest of the process*: although it is likely that *stratum corneum* sheets are constantly being produced at the interface with the living epidermis (simulated as the growth of the substrate in our model), its outermost layers are probably shed (in spite of the deficient desquamation) or abraded at a certain point. Indeed, the full thickness of the *stratum corneum* is usually less than ~300-400μm, suggesting that an additional mechanism acts to keep it from exceeding this value. Moreover, visible marks of abrasion are sometimes observed over the papillae (Supplementary Fig. 8). We did not attempt to incorporate these effects explicitly in our model. Instead, we used the mentioned maximum thickness value of the *stratum corneum* as a 'loose' (*i.e.*, a minimum requirement) stopping criterion for our simulations, as detailed above.

Code availability. The source code is made available, upon publication, on the website of the Laboratory of Artificial and Natural Evolution: https://www.lanevol.org

Analysis of real and simulated geometries

Vertex angle probability density profile estimation. The vertex angles of the patterns of papillae and cracks, on macro photographs of real samples as well as on simulated geometries, were measured using an in-house developed marking toolkit (Supplementary Fig. 13). All photographs, micrographs and screenshots selected for marking were taken "head-on" (*i.e.*, with the camera axis perpendicular to the skin), such that using 2D angles is well-justified. High resolution and magnification images in which cracks or papillae are clearly visible were used. Vertices containing hair or to which more than three cracks converge were excluded from the analysis. In total, roughly 12,000 angles (~4,000 vertices) were measured and used for statistical purposes. We estimated the probability density functions of the distribution of vertex angles of papillae and cracks using a kernel density estimate. This method was preferred to histograms due to its superior performance in terms of statistical bias (*23*). A standard Gaussian kernel was employed, and the bandwidths were selected using the Sheather-Jones "plug-in" approach (*24*) due to its overall good performance in many practical situations (*23*). Confidence intervals were estimated using a Monte Carlo case resampling bootstrapping scheme with 1000 repetitions. Finally, given that the distributions estimated in this work frequently exhibit an unskewed peak centered around ~120°, we used the cumulative probability $P(120°-X < \theta < 120°+X)$, with $X = 10°$-$20°$, as a quantitative measure of the sharpness of this central feature.

Angular scatter plots. Density profiles are known to suffer from a number of shortcomings, most importantly: *(i)* the binning of vertex angles gives an apparent uneven weight to certain types of junctions (a network in which half of the junctions are 'triple-120°' and the other half 'T-junctions' - '90°-90°-180°' - will have 50% more 120° angles than 90° ones), and *(ii)* when networks exhibit a mixture of different junctions types, it is often hard to evaluate the contribution of each and to estimate appropriate statistical measures for the magnitude and direction of the angular spreading. To overcome these difficulties, we exploited the fact that the 2D networks of interest are composed solely of tri-junctions: we start by sorting the three angles of each junction such that $\theta_1 \leq \theta_2 \leq \theta_3$ and, given the constraint $\theta_1 + \theta_2 + \theta_3 = 360°$, we represent each junction by the 2D-point (θ_1,θ_2). Moreover, each point must lie in the region bounded by $0 \leq \theta_1 \leq \theta_2$ and $0 \leq \theta_2 \leq 180 - \theta_1/2$.

In this way, we plot the angular data in a manner that is straightforward, easy to interpret and, most importantly, without losing any information due to binning. We termed this representation 'Angular Scatter Plot' on which we represent the 2D average of the angles θ_1 and θ_2, represented by the point $(\bar{\theta}_1, \bar{\theta}_2)$. We also compute the 2D confidence regions (error ellipses based on the covariance matrix of the data and scaled using chi-squared distribution values) that are visualized as shaded areas on the angular scatter plot and, hence, indicate both the magnitude and the direction of the angular spreading. For visualization purposes, we use low probability confidence levels (~50%) as it helps discerning the orientation of the spreading. Note however that both the orientation and the shape (eccentricity) of the error ellipses are independent of the confidence level. Additionally, to further facilitate the qualitative evaluation of the data, we partition the allowed angular space into three regions (delimited by dashed lines in the plots) obtained by calculating the Voronoi diagram generated by three archetypal junctions: 'triple-120°', '90°-135°-135°' and '90°-90°-180°' ('T-junction'). Finally, we use the square root of the area of the one standard deviation (~68% confidence level) error ellipse, $A_{1\sigma}$, as a quantitative measure of the total angular spreading, σ_θ. Specifically, $\sigma_\theta = (A_{1\sigma}/\pi)^{1/2}$. This is essentially equivalent to the standard practice of using the determinant of the data's covariance matrix as a measure of dispersion.

Trough/Papillae segmentation. To validate quantitatively the results of the physical model and generate meaningful comparisons with measurements on real skin samples, we segmented the simulation mesh into 'trough' and 'papillae' regions by making use of surface curvature. Concretely, we noticed that troughs exhibit a fairly uniform and negative mean curvature (we use the convention that cylinders with outward-facing normals have positive mean curvature), while the remaining regions tend to have positive values (Supplementary Fig. 10A). Thus, by selecting an appropriate mean curvature threshold, a straightforward segmentation of the mesh into 'troughs' and 'papillae' can be achieved. We manually corrected any mistakes of this thresholding routine (Supplementary Fig. 10B).

Supplementary Discussion

Function of the African elephant skin channels

Available research suggests that crevices help the African bush elephant retain water and mud on its skin for improved evaporative cooling, insect protection and shielding from intense solar irradiation (see Main Text). Although studying the adaptive value of the skin pattern is not the main focus of this work, we have independently confirmed *in vivo* the remarkable water retention and spreading properties of the African bush elephant's skin. Indeed, when a localized point on the skin surface is wetted using a syringe (Supplementary Movies 1 and 2), we observe that *(i)* most water is absorbed by the skin, *i.e.*, very little is wasted, *(ii)* water diffuses quickly on the skin (up to ~2-3 cm/s, at the onset of diffusion), and *(iii)* it flows against gravity, suggesting that capillary effects play an important role in the processes of water retention and diffusion. We speculate that the channels' contribution to this phenomenon is two-fold: first, they form a network of narrow (~50µm, Figs. 1C, 2A,B) cracks that spans essentially the whole surface of the animal, and second, they

provide an access for water to the tight spaces in-between *stratum corneum* keratin sheets. Thus, we propose that the *stratum corneum* of the African bush elephant effectively behaves as a porous material, the 'pores' being the ubiquitous network of cracks and inter-sheet spaces, that allow for both the rapid diffusion and the increased retention volume of water. We note that this picture is not incompatible with the observations that the skin of Asian elephants (which lacks cracks but possesses papillae, Fig. 1G) also displays increased water and mud retention capabilities, albeit less pronounced than those of African bush elephants (*25, 26*). Indeed, the troughs between papillae frequently exhibit widths of ~100-150μm, probably well within the range of length scales at which capillary action can overcome gravity, explaining why Asian elephants can also retain water on their skin. However, the African bush elephant's skin cracks are considerably narrower (leading to stronger capillary effects) and potentially allow water to distribute all over the animal's *stratum corneum*, thus increasing the volume that can be retained and buffering evaporation, resulting in longer drying times.

Angular Analysis

One remarkable aspect of the pattern of cracks visible on the skin of the African elephant is the fact that these seem to be mostly confined to the trough regions (although rare exceptions occur; Fig. 5E). Qualitatively, we have attributed this characteristic to the development of strong bending stresses and subsequent fracture of part of the *stratum corneum* in those areas. Being essentially confined to the troughs, cracks usually have very little 'freedom' when it comes to selecting their direction of propagation, except when they approach the intersection of three troughs (the 'vertices' of the pattern of troughs). There, the additional room allows for a heterogeneous stress distribution (see above) which may influence both the direction of further propagation of a given crack and the way in which multiple cracks intersect. To study this effect in a quantitative manner, we have compared the distributions of 'vertex' angles generated by the intersection of cracks with those inferred from the intersection of skin troughs (assuming that the troughs meet at a single point, Fig. 5B). Note that if the zones where troughs intersect were sufficiently small, the angular distributions of troughs and crack vertices would be roughly coincident, as the cracks would have little possibility to reorient themselves. However, our results indicate that the vertex angles of troughs (Supplementary Fig. 14 and Supplementary Table 2) and cracks (Supplementary Fig. 15 and Supplementary Table 3) differ markedly. Concretely, we observe that *(i)* while the angular distributions of both skin features are roughly centered at 120° and symmetric around that value (sample skewness $b_1^{troughs} = -0.0395$ and $b_1^{cracks} = -0.1719$), the probability density profile of trough vertex angles exhibits a considerably sharper peak ($P(110° < \theta < 130°) \approx 0.62$ for troughs *vs* ≈ 0.41 for cracks), and *(ii)* whereas troughs vertices often exhibit a 'triple-120°' angular profile with only mild variation (Supplementary Fig. 16, $\bar{\theta}_1 \approx 108°$, $\bar{\theta}_2 \approx 120°$ and $\sigma_\theta \approx 8.8°$, see 'Materials and Methods' for details), crack vertices exhibit instead a wide variety of configurations ranging from 'triple-120°' to '90°-135°-135°' junctions (Supplementary Fig. 16 and Supplementary Table 4, $\bar{\theta}_1 \approx 100°$, $\bar{\theta}_2 \approx 121°$ and $\sigma_\theta \approx 14.1°$). Interestingly, very few 'T-junctions' (the hallmark of free planar cracking (*27*)) are present on the skin of African elephants (inset of Supplementary Fig. 15A), a consequence of both the restricted crack propagation paths imposed by the troughs and the fundamentally different characteristics of the physical system (a thin shell of material where bending stresses play a crucial role).

Next, we compared the simulated skin cracks with the real ones using the same quantitative metric, vertex angles at the intersections of cracks, and studied how it varies during a typical simulation (Supplementary Fig. 17). Even though cracks form at a fairly steady pace after a certain growth threshold is reached, we noted that they first appear as narrow cracks that meet at a specific point in a given trough junction (Supplementary Fig. 17A). This process continues until almost every trough and junction contains cracks (Supplementary Fig. 17B). Finally, more cracks form close to the preexisting ones, effectively resulting in the widening of trough and junction cracks (Supplementary Fig. 17C). As this 'maturation' process takes place, the angular characteristics of the pattern change considerably: the central peak of the probability density function sharpens ($P(110° < \theta < 130°)$ increases from ≈ 0.27 to ≈ 0.41) and the junctions' profile continuously shifts towards the 'triple-120°' configuration (Supplementary Fig. 17D,E). This transition from an 'immature' to a 'mature' cracking pattern seems to occur for all the tested simulation parameters, following roughly the same trajectory (Supplementary Fig. 18). The angular characteristics of simulated mature cracks fit those of real skin cracks substantially better than immature simulated cracks (Supplementary Fig. 19), *i.e.*, the process of maturation drives the profile of simulated cracks towards the one observed on the skin of the animal. Note that a certain degree of maturation is likely to occur in the real system because one expects the *stratum corneum* to continuously grow and the desquamation rate to be roughly constant. Testing this hypothesis with direct observations would require following the development of cracks in an individual since its birth (newborn individuals do not exhibit cracks, see below).

<u>Evidence for cracking as the generative mechanism of the African elephant skin channels</u>

We give below a detailed account of both the direct and indirect evidence that we have acquired in support of the hypothesis that the intricate pattern of channels visible on the skin of the African elephant (Fig. 1C) are formed through a process of bending fracture occurring on the outermost layers of the animal's *stratum corneum*.

Visual aspect of the structures. The pattern of cracks on the skin of the African elephant lies on top of an uneven substrate composed of the animal's dermo-epidermal papillae (Fig. 1E,F). Given that a substrate's shape can affect the appearance of cracks above it (*28*), papillae are expected to greatly influences the overall organization of the elephant crack skin pattern, including, *e.g.*, the cracks' paths and the distribution of edge angles at vertices (points where three cracks meet, see *'Angular Analysis'*). In spite of this key particularity, the network of cracks found on the skin of juvenile and adult African elephants still shares some characteristics with the patterns visible on canonical cracking systems on flat substrates (*e.g.*, dried mud/clay or damaged asphalt), such as irregularly shaped domains, hierarchical attributes and incomplete edges (Supplementary Fig. 12A-C). Additionally, we note that in some cases the cracked pattern on the skin of the animal bears a strong resemblance to the quasi-hexagonal organization produced by an advancing drying/cooling front (*29*), observed, *e.g.*, in starch experiments (inset of Supplementary Fig. 12D). However, this similarity should be ascribed to the particular organization of the papillae forming the substrate rather than to the cracks *per se*, given that the latter simply follow the troughs of the former. Note that investigating the process involved in the patterning of the papillae is beyond the scope of this work as it would require analyses of

embryological series (papillae are already present in newborn individuals; Supplementary Fig. 4).

Light and electron microscopy of a crack. Light and electron imaging of cracked regions of the animal's *stratum corneum* (Fig. 2A,B) reveal a fracture characterized by *(i)* a clear alignment between the layer's sheets on both sides of the discontinuity (the same alignment is visible also in unbroken samples; Fig. 2C), and *(ii)* little or no recoil, consistent with the hypothesis that the cracks form mainly due to flexural stresses. These two features are reproduced by our numerical simulations (Fig. 5G-I; see *'Numerical Simulations'*). Moreover, we found that the fracture propagates only down to a certain depth, leaving the innermost keratin sheets of the *stratum corneum* intact, in agreement with the results obtained from the mechanical removal of the cracked layer (see below). Qualitatively, this observation is explained by the expected higher hydration of the deep layers of the *stratum corneum*, which translates into higher critical strains (*16, 18*).

Mechanical removal of the stratum corneum. When the *stratum corneum* from fixed African elephant skin samples is mechanically removed, we observe that it generally comes off as a single patch of material delimited by cracks (Supplementary Movie 3) although, in some cases, a patch with cracks inside it can be extracted at once (Supplementary Movie 4). These results are consistent with the morphological picture obtained using light and electron microscopy: since a crack is a zone where most (but not all) of the keratin sheets are broken, the *stratum corneum* will preferentially detach in these areas when subject to external stresses. However, the fact that the innermost sheets of the *stratum corneum* are continuous even in cracked regions (Fig. 2A,B) makes it possible to extract patches that have cracks inside them.

Crack reorganization following the regrowth of the stratum corneum. Since new sheets of *stratum corneum* are constantly being produced by the skin and older ones are abraded or shed (in spite of the deficient desquamation), one expects that the pattern of cracks might reorganize during these cycles. To test this, we followed *in vivo* a patch of skin on the animal from which the *stratum corneum* had been initially extracted by natural abrasion then mechanically 'cleaned up' by removal of additional material using tweezers (Supplementary Fig. 1). The regrowth of the *stratum corneum* during a period of 225 days was accompanied by a number of small-scale pattern rearrangements, such as the 'healing' of some cracks, the widening of others or the clumping of several papillae under the same uncracked domain (Supplementary Fig. 2). On the other hand, the broad-scale geometry of the pattern remained largely unaltered as many cracks failed to close. This result that can be qualitatively understood by noting that the *stratum corneum* in cracked areas is connected by a smaller number keratin sheets, hence, we expect it to be more fragile and prone to re-cracking there, consistent with the findings of our *stratum corneum* removal experiments on fixed skin samples (see above). Finally, we point out that a similar phenomenon has been observed in mud or clay cracks when subject to repeated wetting/drying cycles (*30*).

Trough widening on cracked samples. When the *stratum corneum* is mechanically removed from fixed skin samples, we observe that the spacing of the troughs between papillae is not homogeneous (Supplementary Fig. 3A,B). Moreover, we note that *(i)* the distribution of trough width is not random but instead tends to form connected 'lines' on the underlying skin, and *(ii)* a wide trough is almost always associated with a cracked region of the *stratum corneum* (the converse, however, is not true). Employing a semi-

quantitative approach, we show here that this observation is consistent with the cracking hypothesis coupled with the normal growth of the animal. As mentioned above, the process of *stratum corneum* regrowth occurs on a timescale of hundreds of days (Supplementary Fig. 1). One can thus expect that the animal itself will also grow during this period, particularly young specimens. However, due to the African elephant's deficient desquamation, the outer/older sheets of *stratum corneum* are not shed, and since these are composed of dead tissue that does not experience growth, they will oppose the widening of the underlying troughs. If, on the other hand, the *stratum corneum* in a trough is already cracked, this resistance does not exist and the trough can widen more freely. This qualitative picture can be made more quantitative by considering a simple 'toy model' in which two adjacent papillae are represented by blobs connected by springs (Supplementary Fig. 3C). The mechanical constraints of the *stratum corneum* and of the rest of the skin are separated into two different springs with constants k_{SC} and k_{skin}, respectively. Moreover, we assume that before growth starts both springs have the same rest length, L_0. Once the animal starts growing, however, the rest length of the 'skin spring' changes to $(1+g)L_0$, where g is the relative growth, and the separation distance between the two papillae, *d*, will now be given by the formula

$$\frac{d}{L_0} = 1 + g\,\frac{k_{skin}}{k_{skin}+k_{SC}}.$$

While we cannot accurately estimate the values of k_{SC} and k_{skin}, we can nevertheless consider two important limits: if, for a specific trough, the *stratum corneum* is broken, we can expect the corresponding spring to be very compliant or altogether absent. In that case, $k_{SC} \approx 0$ and $d \approx (1+g)L_0$, that is, the trough widens with the growth. On the other hand, if the *stratum corneum* is intact, then $k_{SC} > 0$ and the widening is reduced (Fig. S3C). Additionally, since the partially dry *stratum corneum* is considerably stiffer than the rest of the skin, it is likely that $k_{SC} >> k_{skin}$, in which case $d \approx (1+g\ k_{skin}/k_{SC})L_0$, *i.e.*, the relative increase in trough width is curtailed by a factor k_{skin}/k_{SC}. Finally, we note that some cracks being not associated with widened troughs is not inconsistent with this model: they may have appeared more recently, and thus the corresponding troughs have not yet experienced the effects of the animal's growth. This possibility is corroborated by our numerical simulations which indicate that cracks tend to form in a progressive manner rather than in an abrupt cascade (see 'Numerical simulations').

Absence of cracks on the skin of newborn individuals. Unlike juveniles and adults (Fig. 1 and Supplementary Fig. 12), newborn African elephants do not exhibit a visible cracking pattern on their skin (Supplementary Fig. 4). This striking observation is fully consistent with the cracking hypothesis. Indeed, the formation of cracks in the *stratum corneum* requires that *(i)* the outermost part of that skin layer is sufficiently dry to allow for brittle fracture at a low critical strain, and *(ii)* a substantial thickening of the skin layer has occurred, allowing considerable stresses to develop. It is clear that the first condition is not satisfied while the animal is inside its mother's womb and its *stratum corneum* is probably fully hydrated. Moreover, it is likely that little *stratum corneum* thickening occurs before birth, further reducing the likelihood that the conditions for cracking are met prenatally. Thus, the absence of cracks on the skin of newborn individuals is to be expected and is confirmed by our observations, lending further support to the proposed patterning mechanism (cracking).

Histological and morphological similarities with ichthyosis vulgaris. African elephant skin sections stained with hematoxylin and eosin (Figs. 3 and Supplementary Fig.

5) reveal an epidermis exhibiting *(i)* orthokeratosis (hyperkeratosis without nuclei retention in the *stratum corneum*), and *(ii)* no sign of keratohyalin granules, a feature that in humans correlates almost perfectly with the absence of a granular layer (*31*). The same histological characteristics are often observed in patients with *ichthyosis vulgaris*, a common skin disorder believed to be the result of mutations to the gene encoding profilaggrin and that causes dry, scaly and cracked skin in humans (*32*). In particular, the total absence of the granular layer (as opposed to a mere reduction in size) is a trait that seems specific to a subtype of *ichthyosis vulgaris* (*31*) for which patients often exhibit abnormal desquamation (*33*). These considerations suggest that there is a parallel between the skin of African elephants and that of humans affected by *ichthyosis vulgaris*. One can thus speculate that the propensity for dry, brittle skin exhibited by the latter is also present in the former, a condition that would greatly facilitate the formation of cracks in the *stratum corneum*.

Agreement with cracking simulations. To test the proposed mechanism for the formation of cracks in the *stratum corneum* of African elephants, we implemented *in silico* a physical model (see *'Numerical Simulations'* section below) of the mechanical response of the outer sheets of *stratum corneum* when subject to the stress induced by the growth of their underlying counterparts, *i.e.*, assuming that the visible skin pattern arises due to the cracking of the outer *stratum corneum*. We found the results of these simulations to be in good agreement with qualitative and quantitative features of the real system (Figs. 4 and 5). Moreover, this is achieved without resorting to parameter fitting as the model contains no 'free' constant. We interpret this compatibility between observed and simulated patterns as corroboration for the hypothesis that the channels visible on the skin of the African elephant are bending cracks.

Alternative patterning mechanisms

Despite that all our experimental observations and numerical simulations suggest that cracks appear mainly due to the large bending strains that develop in the troughs of a network of skin papillae, two alternative processes could potentially generate the pattern of cracks: *(i) stratum corneum* desiccation, and *(ii)* biological or chemical differences between troughs and papillae. Regarding the former, although previous studies indicate that the in-plane strain due to *stratum corneum* desiccation might be as small as 0.005 (*16*), and thus probably insufficient to cause material failure, one could argue that much larger (~0.2-0.3) in-plain strains could generate the same pattern. To test this hypothesis, we used our numerical model to simulate the shrinkage (drying) of the *stratum corneum*. The resulting pattern is largely incompatible with the crack pattern observed on real elephants, as cracks systematically propagated perpendicular to the troughs (instead of along them) and over the papillae (Fig. 4D). Additionally, cracks did not intersect at trough junctions and displayed the marked recoil typical of desiccation cracks. Thus, these results suggest that *stratum corneum* desiccation plays at most a minor role in generating the pattern of cracks visible on the skin of the African elephant.

On the other hand, we cannot exclude the existence of biological/chemical/material differences between papillae and troughs and that this factor would help confine the cracks to the troughs. Indeed, an earlier study (*34*) suggests an histological and compositional demarcation between trough and papillary regions. Although we were unable to observe such a demarcation (Supplementary Fig. 5), it is conceivable that additional biological or chemical cues may participate in establishing the pattern of cracks on the skin. Nonetheless,

we emphasize that our physical model can reproduce most of the features of the African elephant real skin pattern without resorting to any form of bias, suggesting that the contribution of the latter for crack pattern formation may be small, if any.

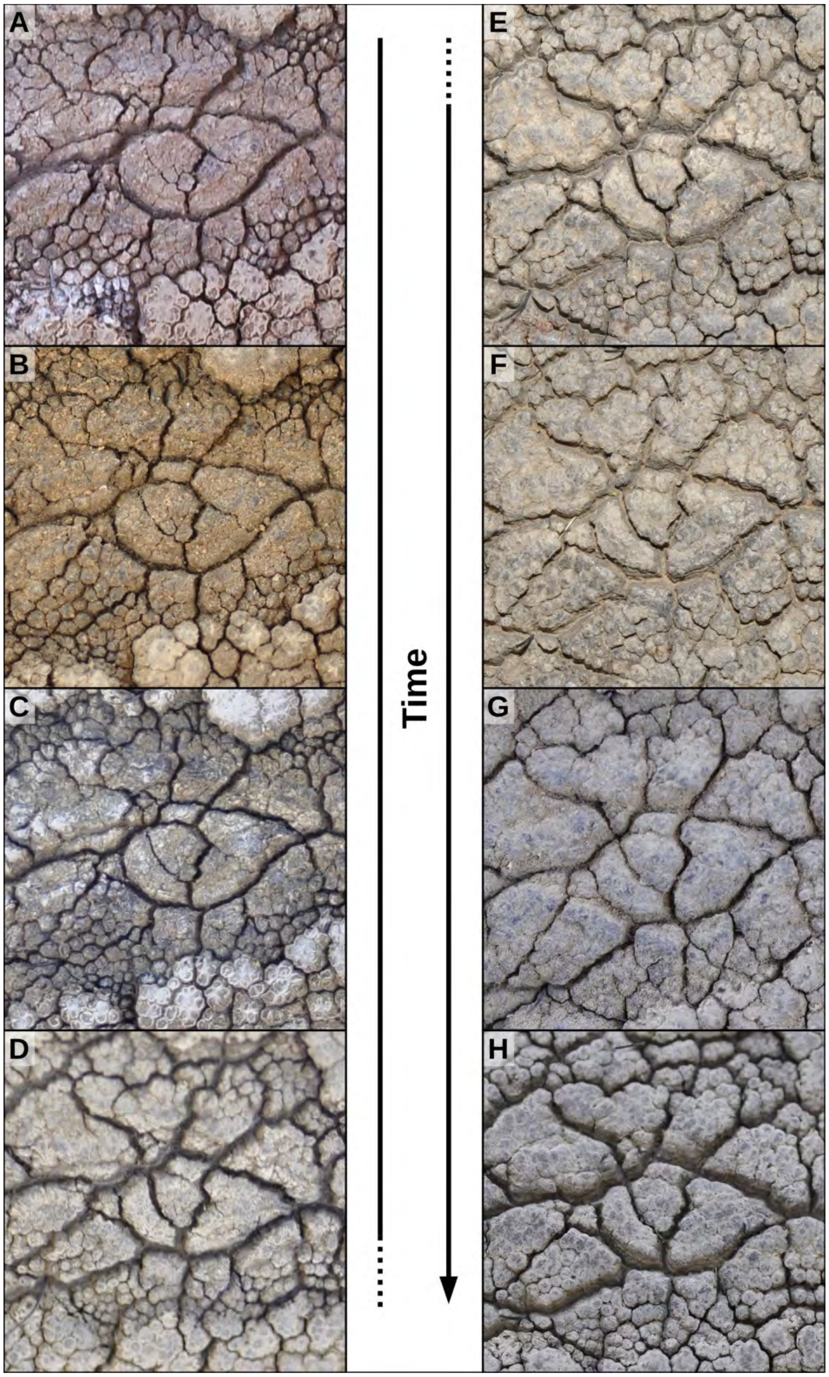

Supplementary Figure 1. ***In vivo*** **regrowth of the** ***stratum corneum*****.** A patch of skin situated on the buttocks of the animal, and from which the *stratum corneum* had been partially extracted (through both natural abrasion and mechanical cleaning with tweezers), was followed for a period of 225 days, during which a substantial regrowth took place. Several fine-scale pattern rearrangements are visible. Photos taken in 2016 on (**A**) February 3; (**B**) February 22; (**C**) March 5; (**D**) May 31; (**E**) July 8; (**F**) July 25; (**G**) August 16; (**H**) September 16.

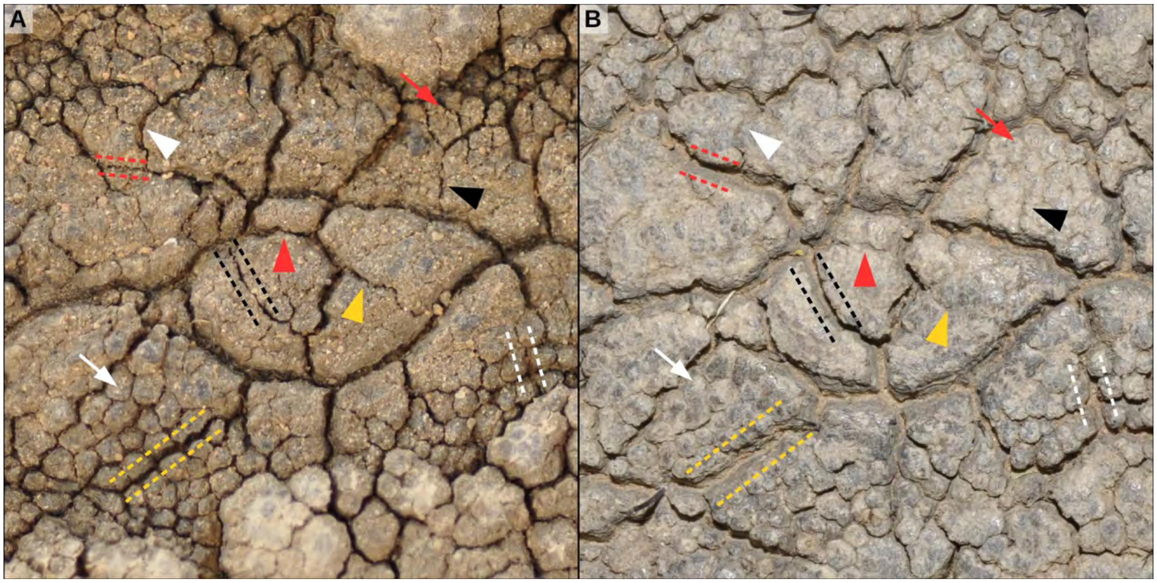

Supplementary Figure 2. Regrowth of the *stratum corneum* leads to a fine-scale reorganization of the pattern. A patch of skin from which the *stratum corneum* had been partially extracted was followed *in vivo* for several months. Although the broad features of the pattern remain unaltered, a number of rearrangements occur, such as the 'healing' (arrowheads) or widening (dashed lines) of some cracks, or the grouping of several papillae (arrows). Photos taken on February 22, 2016 (**A**); and about 150 days later (**B**; July 25, 2016).

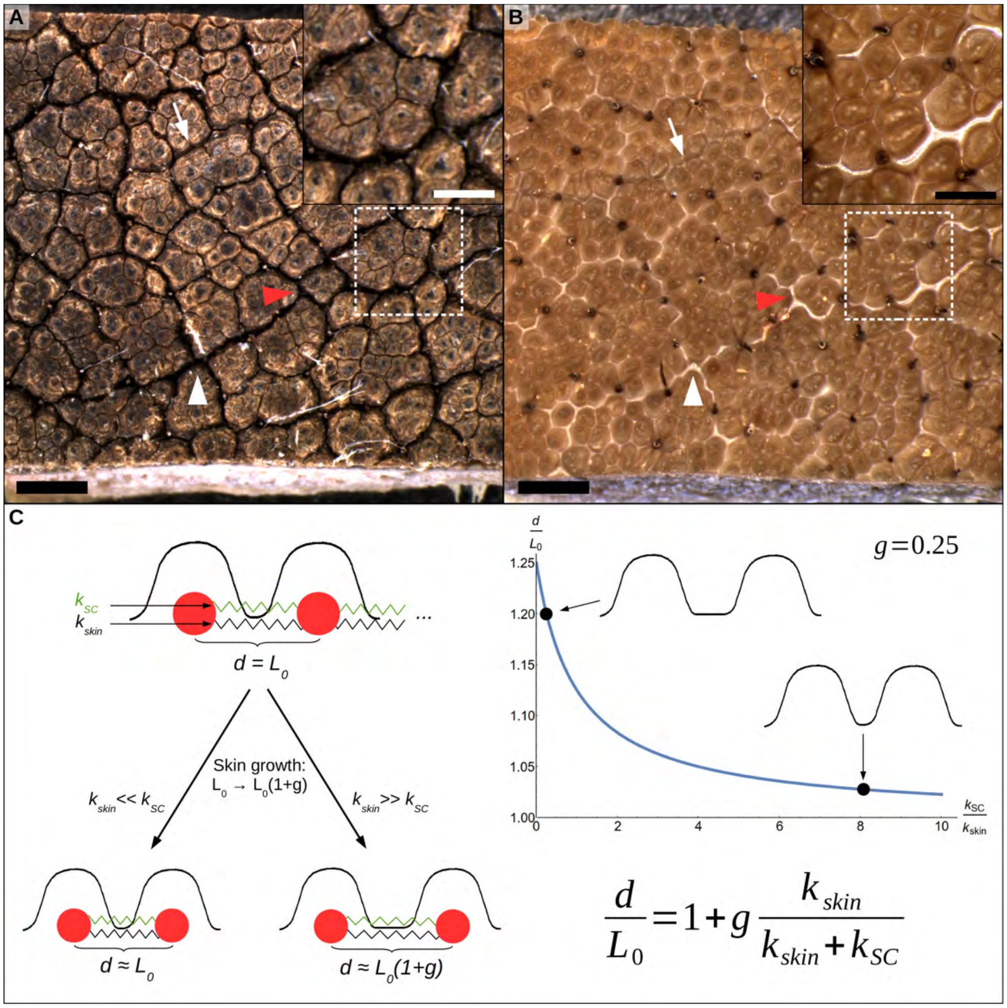

Supplementary Figure 3. Heterogeneous distribution of trough width in young animals. Following the mechanical removal of the *stratum corneum* on a formalin-fixed sample from the forehead of a 1-year-old African elephant (**A**), a heterogeneous distribution of the width of troughs separating the papillae of the underlying skin layers is revealed (**B**). Insets: details of regions in (A) and (B) highlighted by dashed squares. Wider troughs are almost always associated with cracked regions (arrowheads), although the contrary is not true (arrows). These observations can be explained using a simple semi-quantitative model incorporating only cracking and the animal's growth (**C**; see supplementary Discussion for details). Scale bars: (A) and (B), 1mm; insets, 0.5mm.

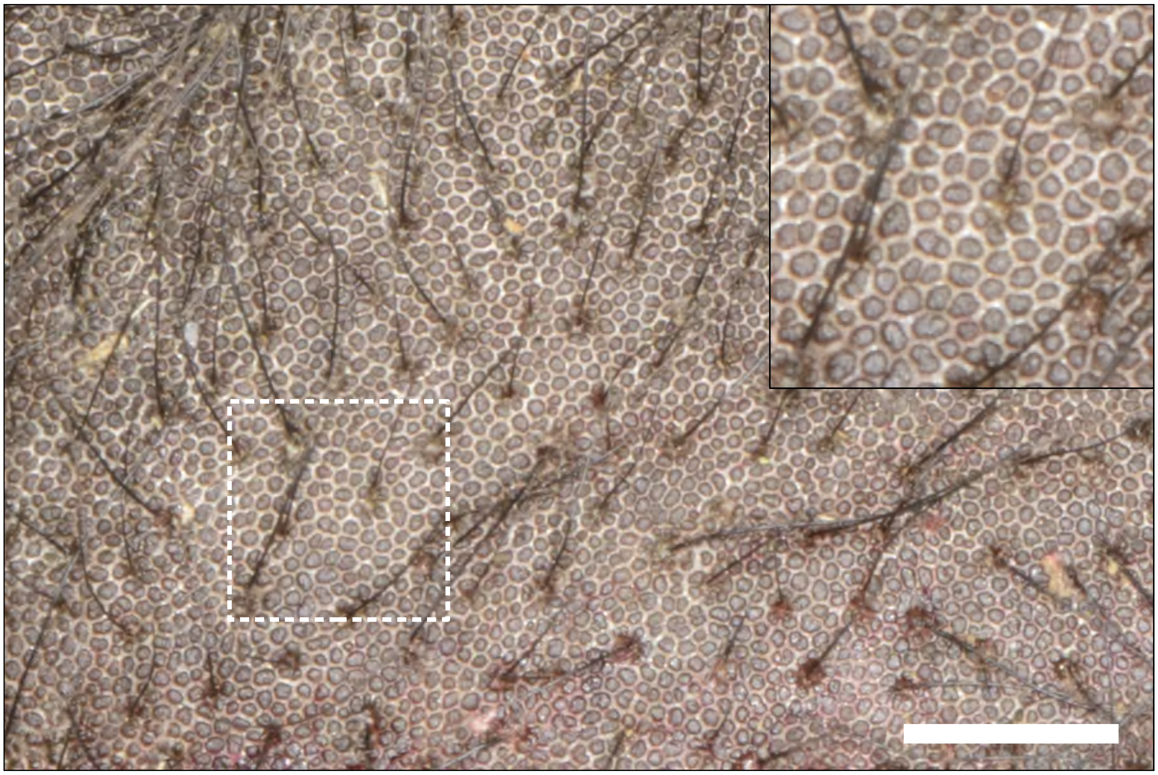

Supplementary Figure 4. Absence of cracks on the skin of newborn African elephants. Unlike juvenile and adult individuals, the skin of newborn African elephants appears to lack cracks. This is consistent with the proposed model for the formation of these structures (see supplementary Discussion). Photograph taken on the flank of the animal less than 3 hours after its birth. Inset: magnified view of the region highlighted by the dashed line. Scale bar: 5mm.

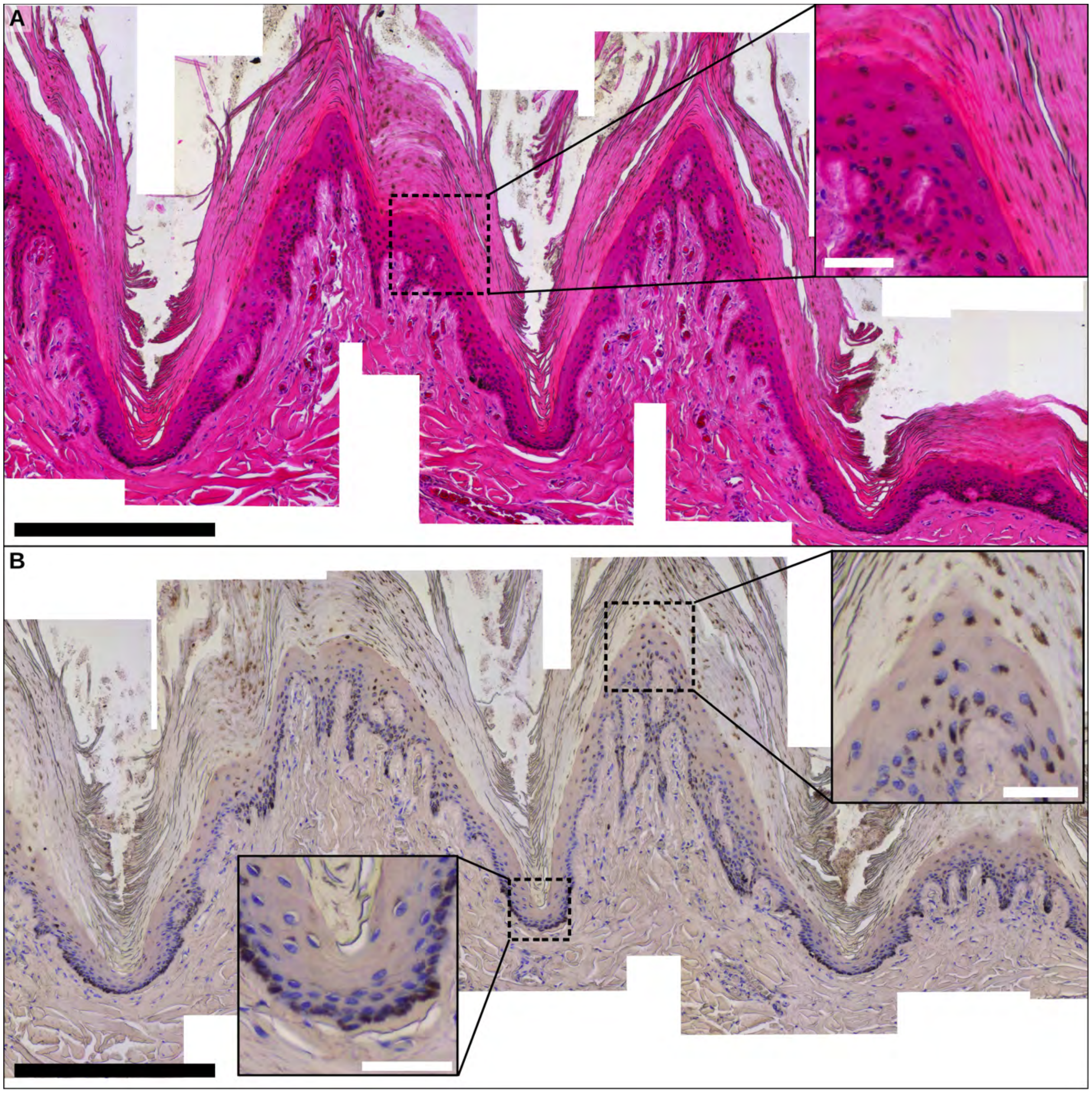

Supplementary Figure 5. The epidermis of African elephants exhibits orthokeratosis and lacks keratohyalin granules. Skin sections stained with hematoxylin (A,B) and eosin (A) reveal a complete absence of keratohyalin granules and a thick but otherwise normal-looking *stratum corneum* (in which the dark spots are melanin pigment and not nuclei). Similar morphological features are generally observed in patients with *ichthyosis vulgaris* (see supplementary Discussion). The images shown are a composition of several (~12) individual focus-stacked pictures (see Materials and Methods) and depict the ear region of an adult African elephant. A similar morphology is found in other body locations. Insets: magnified views of the regions highlighted by the dashed squares. Scale bar: (A) 400µm. Inset: 50µm; (B) 400µm. Insets: 50µm.

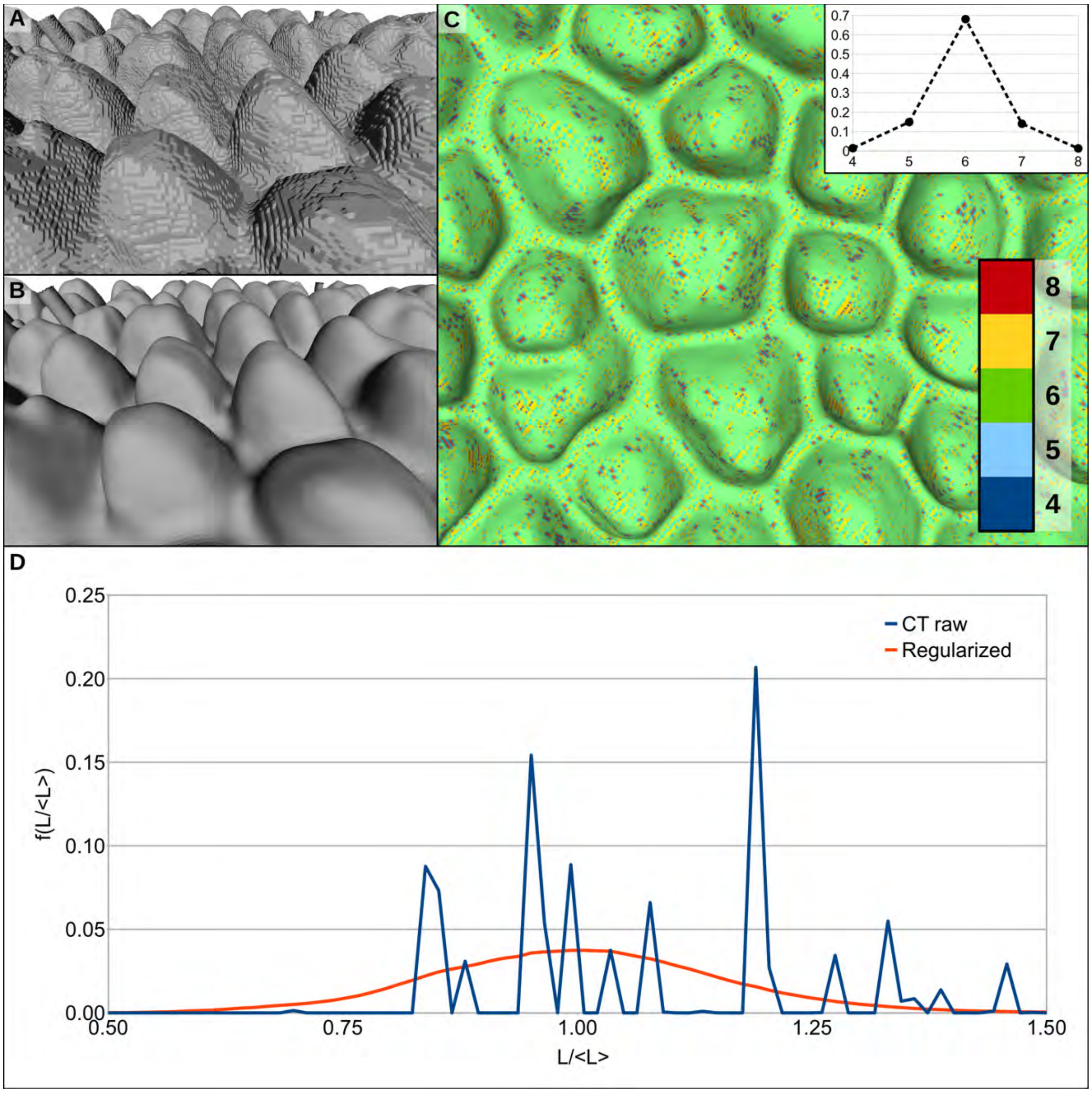

Supplementary Figure 6. Mesh statistics and regularization. The surface extracted from the CT data (A) is rough and exhibits a very irregular edge length distribution (blue line in D), making it ill-suited for our simulations. Hence, we have devised a custom algorithm to smooth out the fine heterogeneities while keeping the large-scale features intact (B). The procedure strongly regularizes the edge length distribution (red line in D). (C) Additionally, the mesh connectivity (number of neighbors per node) is sharply peaked at 6 (inset): ~70% of the nodes have 6 neighbors, showing that the mesh has an approximately hexagonal packing; mesh's nodes have been colored according to their local connectivities: from dark blue for 4, to red for 8 (the mesh contains no internal nodes with less than 4 or more than 8 neighbors). (D) <L> is the mean edge length, while *f(L/<L>)* is the probability density function of the edge length distributions.

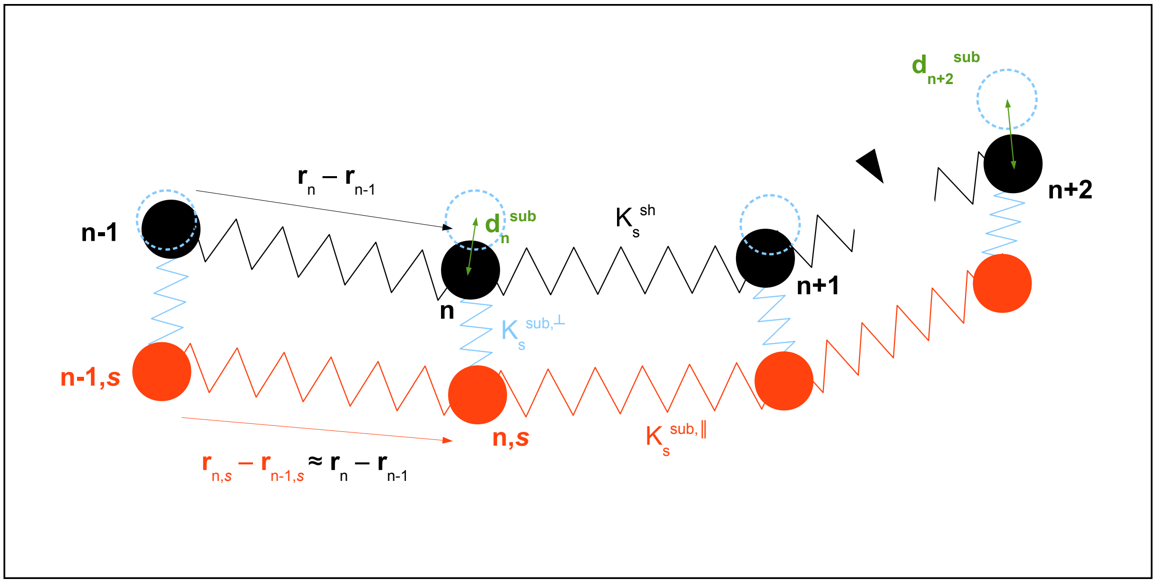

Supplementary Figure 7. Schematic representation of the shell-substrate interaction in our model. We consider two types of substrate effects: in-plane (red springs) and out-of-plane (blue springs). Simulation of the former is greatly simplified by noting that the substrate nodes (red dots) essentially coincide with their shell layer counterparts (black dots), given that these structures are connected. Thus, any vector joining adjacent substrate nodes (red arrow) is well approximated by the vector joining the corresponding shell nodes (black arrow), and the two springs can be assumed to be parallel, *i.e.*, their combined response can be modeled as a single spring with stiffness $K_s^{sh}+K_s^{sub,\parallel}$. On the other hand, the out-of-plane component is simulated as an 'oriented' spring (*i.e.*, one favoring both a specific rest length and a given direction). As such, each shell node will have a unique rest position with respect to the substrate (dashed blue circles), and the restoring force felt by the node will be proportional to the displacement from that position, d_n^{sub}. We assume that only the shell layer's springs (black) can crack (arrowhead).

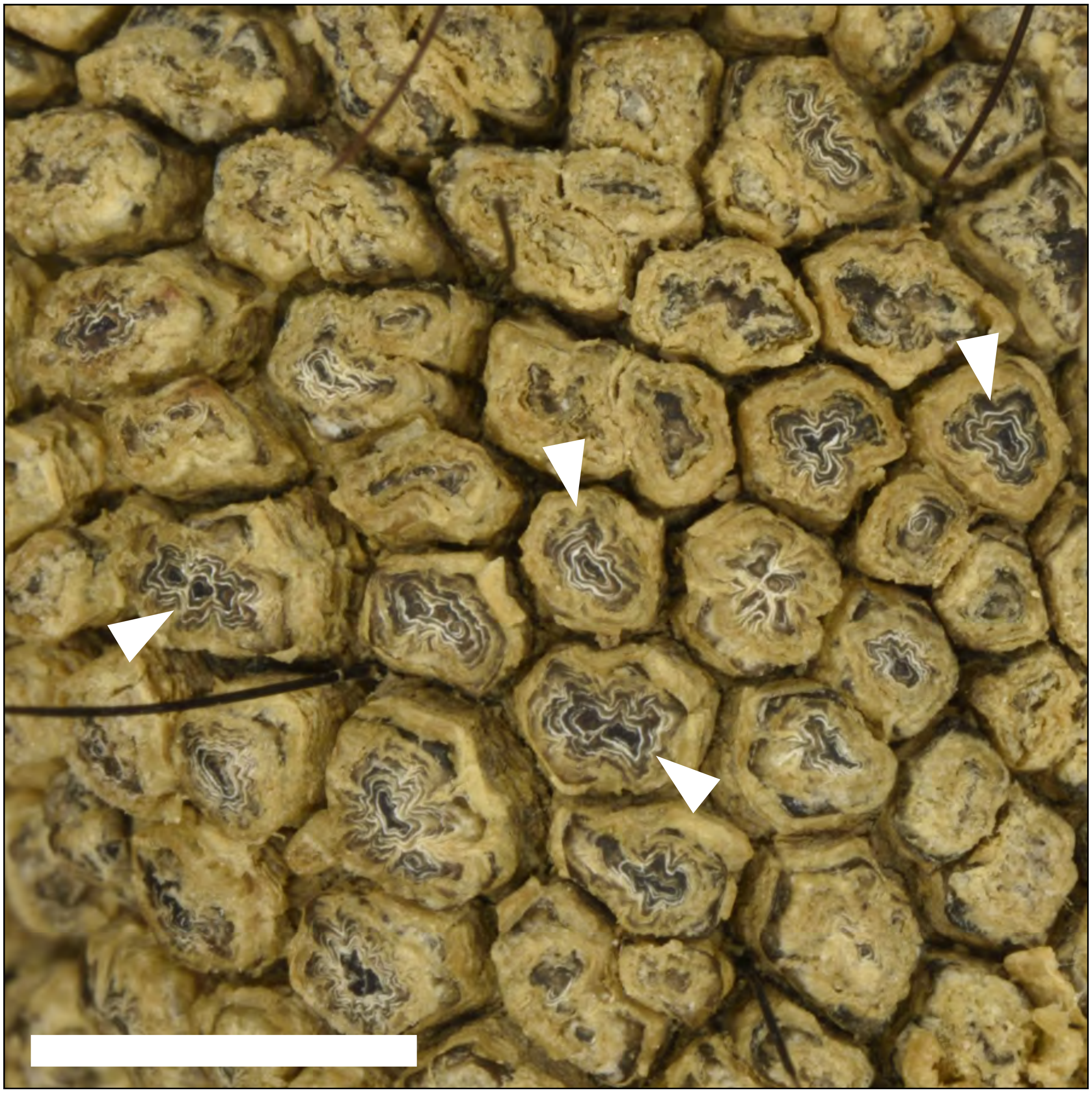

Supplementary Figure 8. Signs of *stratum corneum* abrasion over the papillae. Abrasion, here inferred from the roughly concentric contours of keratin sheets visible over the papillae (arrowheads), may help the animal get rid of the outermost layers of its *stratum corneum*, thus participating in the regulation of the latter's thickness. Scale bar: 5mm.

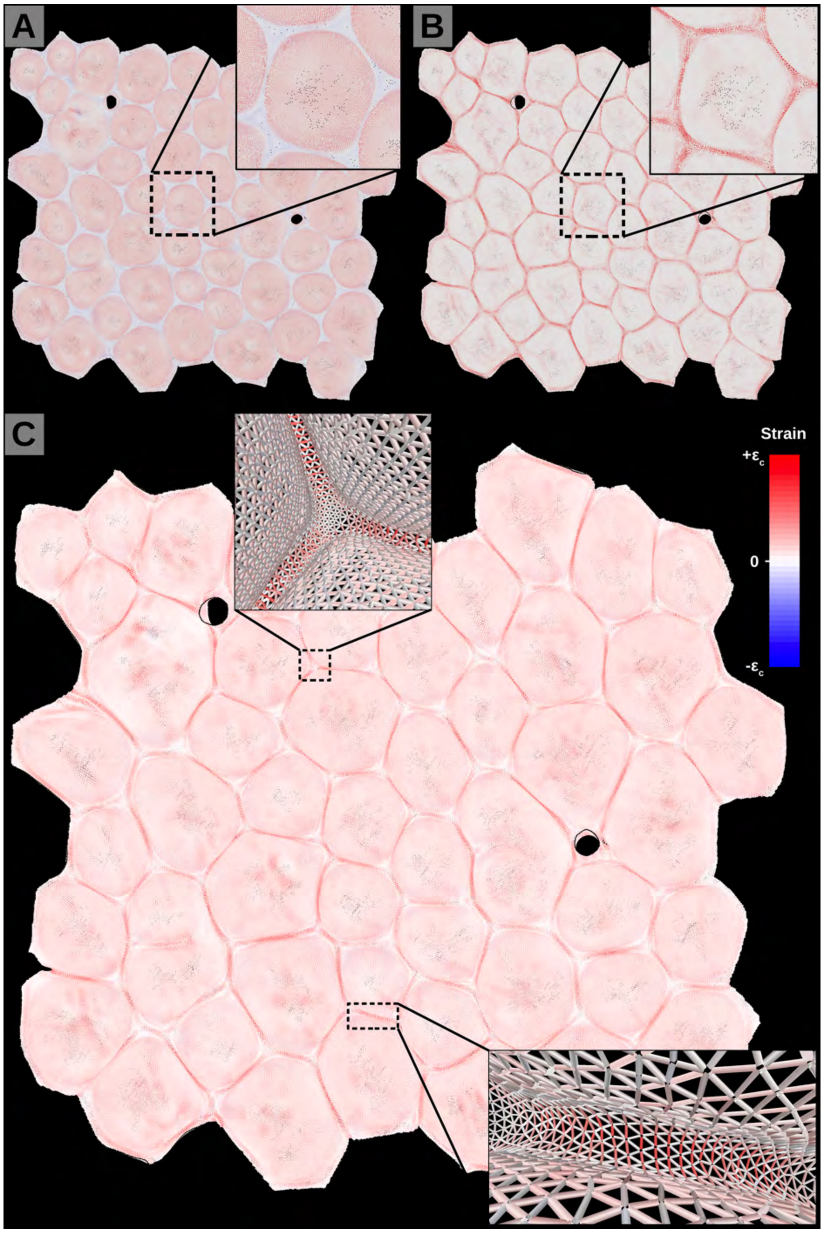

Supplementary Figure 9. Simulated strain distribution on the outer *stratum corneum*. The growth of the underlying (inner) *stratum corneum* coupled with the deficient desquamation of that skin layer lead to the development of both stretching (**A**) and bending (**B**) strains (and stresses) on the outer *stratum corneum* (which we model as a thin shell). The stretching strain is, in general, weakly compressive in the troughs and tensile over the papillae, while the bending strain is essentially confined to the troughs (insets). The full strain (sum of the stretching and bending terms; **C**) is usually tensile all throughout, peaking in the troughs along the direction perpendicular to them (bottom inset in C). The trough junctions exhibit a heterogeneous strain distribution (top inset in C), likely responsible for the different angular characteristics observed in crack and trough junctions. Note that the color map scaling used in (A) and (B) differs from that of (C).

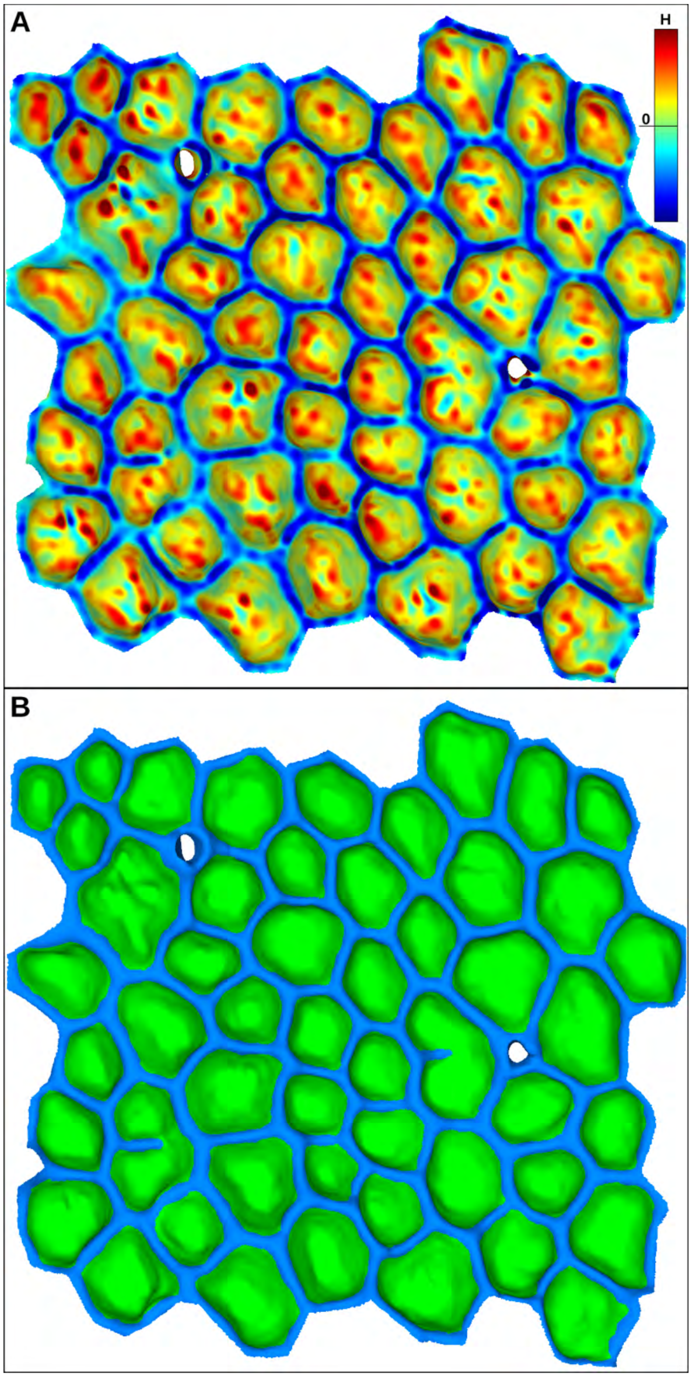

Supplementary Figure 10. Trough/papillae segmentation. (**A**) While troughs exhibit a roughly uniform and pronouncedly negative mean curvature, *H*, the remaining regions of the skin have a much more heterogeneous (but mostly positive) curvature distribution. (**B**) This allows for a straightforward segmentation of the mesh into troughs (blue) and papillae (green). Note that the mesh was generated from the micro-CT scan of a patch of African elephant skin from which the *stratum corneum* had previously been extracted.

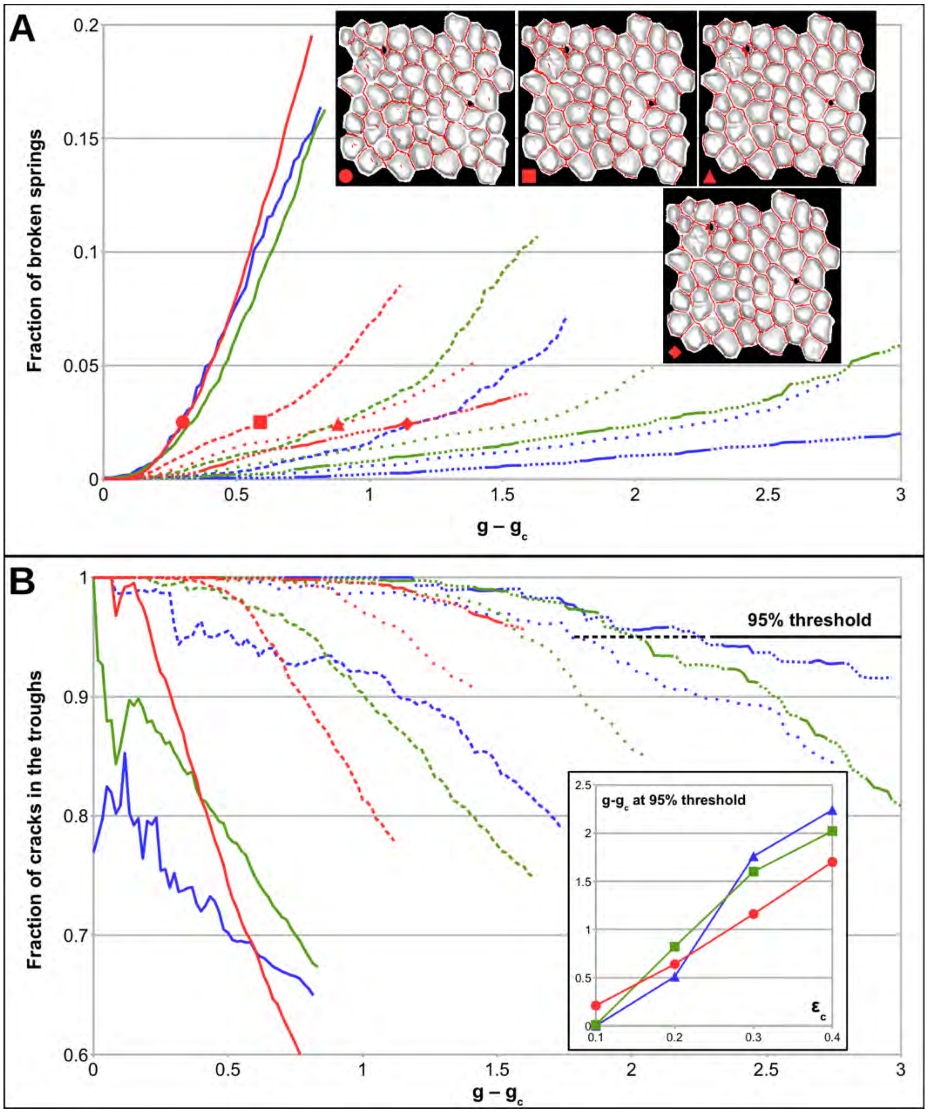

Supplementary Figure 11. Cracks appear progressively and predominantly in the troughs. During growth of the underlying *stratum corneum*, cracks tend to appear in the outer *stratum corneum* at a steady pace rather than in an abrupt cascade (**A**). Moreover, these cracks are essentially confined to the troughs (**B**), particularly for simulations with critical strain >0.1. In most cases, when the fraction of broken springs (cracked mesh edges) reaches 2.5-3.0%, the troughs are already substantially cracked (insets of A, corresponding to the circle, square, triangle and diamond in the main figure). The higher the critical strain, the longer cracks are confined to the troughs (inset of B). The growth of the inner *stratum corneum* (*g*) is expressed in units of average trough spacing, <*d*> (≈125-150μm), and normalized to the growth at which the first crack appears, g_c. Line style legend: in (A) and (B), solid lines stand for simulations with critical strain (ε_c) = 0.1, dashed for ε_c = 0.2, dotted for ε_c = 0.3 and solid-dotted for ε_c = 0.4. Red, green and blue (also in inset of B) indicate simulations with *h*/<*d*> = 1/6, 1.5/6 and 2/6, respectively, where *h* is the thickness of the outer *stratum corneum*.

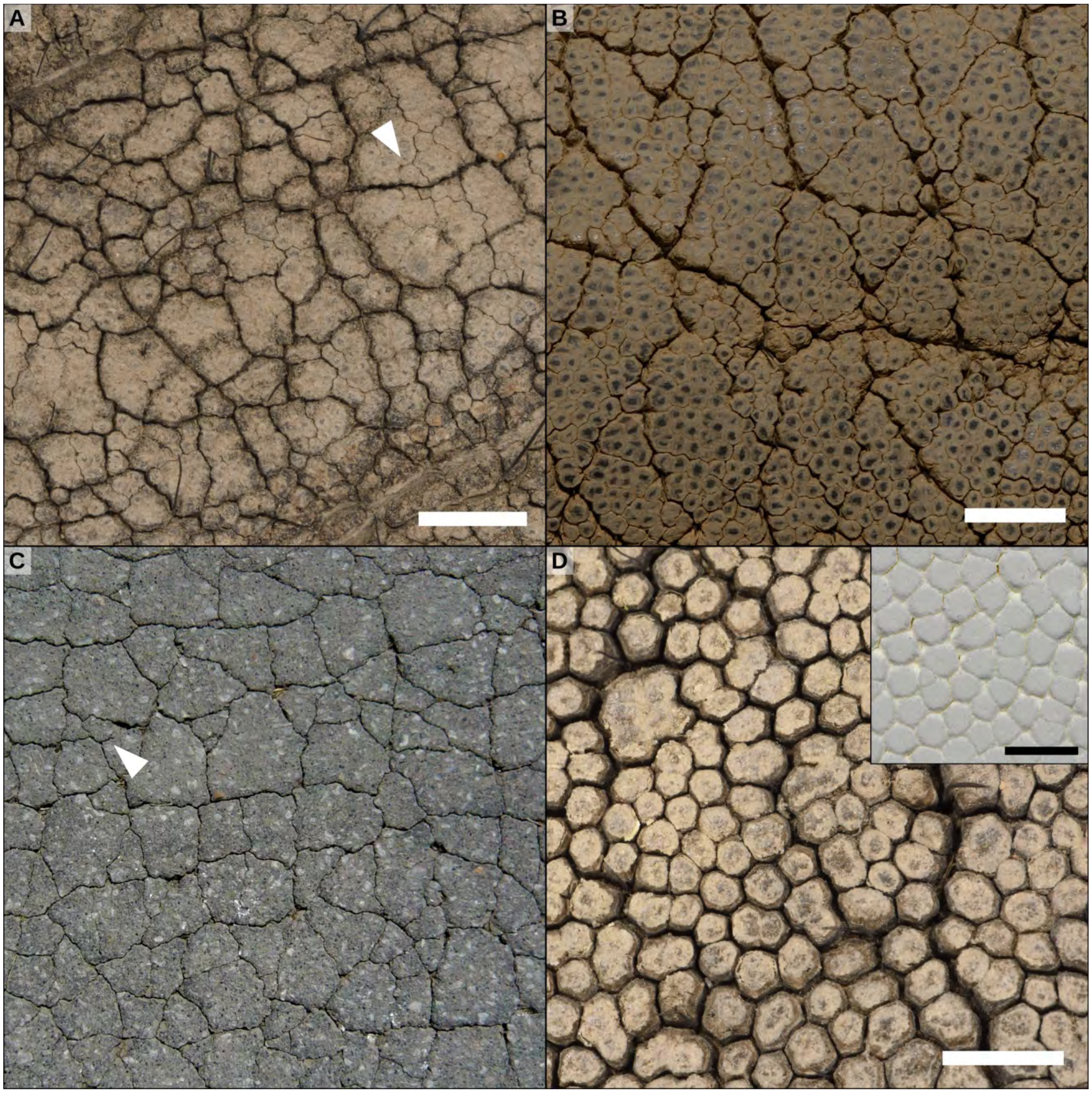

Supplementary Figure 12. Visual comparison between the African elephant's skin morphology and other patterns formed through physical cracking. Even though the cracks on the skin of the elephant (A,B,D) lie on top of a complex-shaped substrate (*i.e.*, the dermo-epidermal papillae), they still exhibit visual similarities with canonical cracking systems such as, *e.g.*, damaged asphalt (C) or dried mud. Similar features include irregularly shaped domains, hierarchical attributes (inferred from the differences in crack width) and incomplete edges (arrowheads in A and C). In some instances (D), the pattern of cracks on the African elephant skin resembles that of starch experiments (inset in D), although in this case the comparison is misleading (see supplementary Discussion). Scale bars: (A), 10mm; (B), 5mm; (D), 5mm; Inset, 5mm.

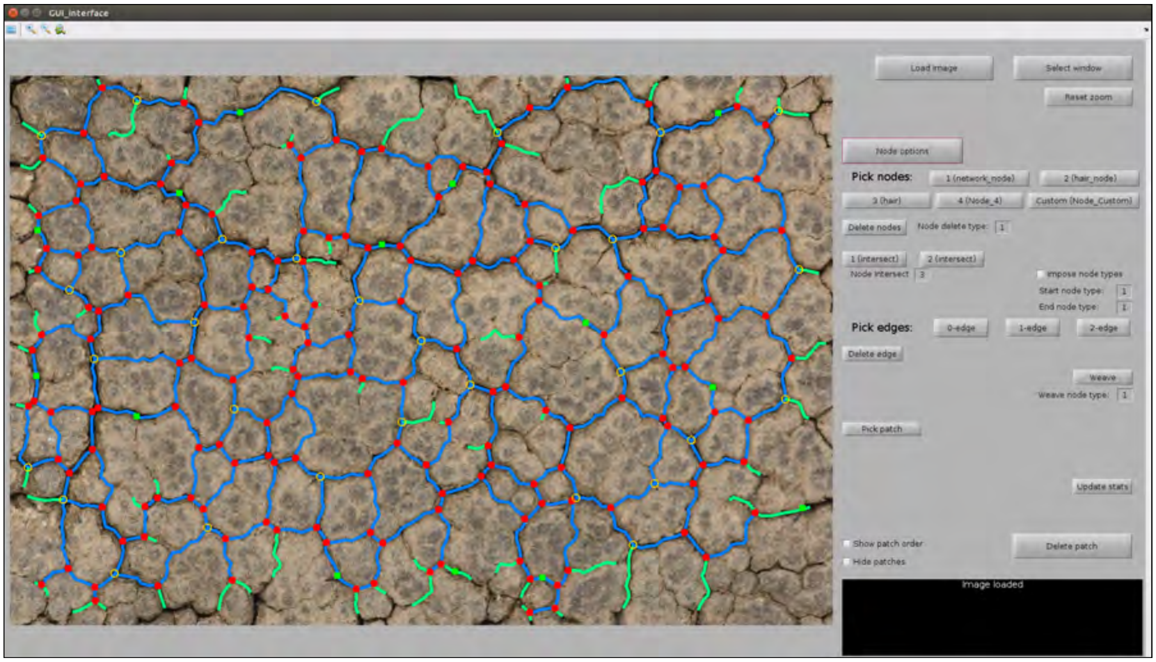

Supplementary Figure 13. A custom graphical user interface and in-house implemented MATLAB program were used to measure the vertex angles of the networks of papillae and real/simulated cracks (pictured is the forehead of an adult animal). All the cracks (blue and green lines) and hairless vertices (red dots) were manually marked. Vertices containing hair (yellow circles) were excluded from the angular analysis.

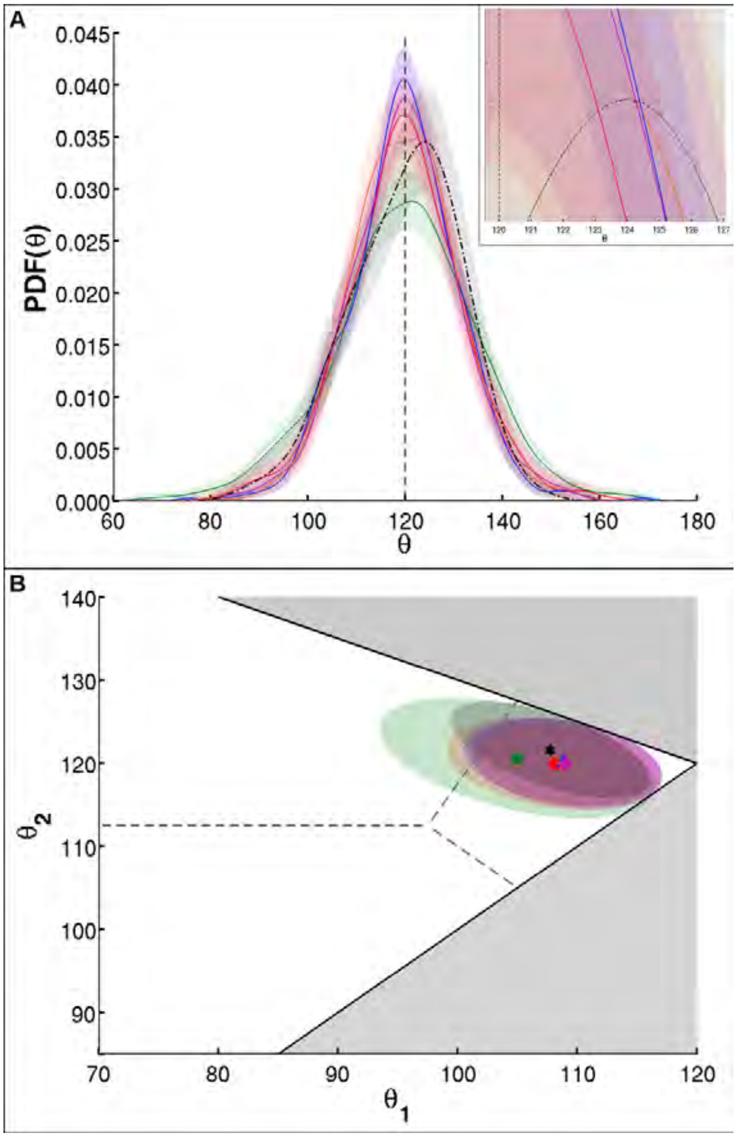

Supplementary Figure 14. Trough junctions exhibit an angular profile with a strong 'triple-120º' component. The probability density function (PDF) of vertex angles at trough junctions (**A**) displays a sharp non-skewed peak (>90% of the angles fall between 100º and 140º) centered close to 120º. The 2D angular scatter plots (**B**, and see Methods) of the same samples similarly reveal that most vertices lie inside the 'triple-120º region'. Moreover, they exhibit relatively low angular spreading (average σ_θ = 8.8). The angles were measured using micrographs of African elephant skin from which the *stratum corneum* had been previously removed (solid lines in (A), circle, square, diamond and triangles in (B)), or a screenshot of the mesh used for simulations (dot-dashed line in (A), black star in (B)). The latter exhibits an angular profile that is skewed and off-centered by ~4º (A, inset), likely the result of statistical fluctuations as the number of junctions analysed in this case is small (70). Shaded regions represent 95% confidence intervals in (A) and 50% confidence regions in (B); see Supplementary Methods. Number of data points are given in Supplementary Table 2.

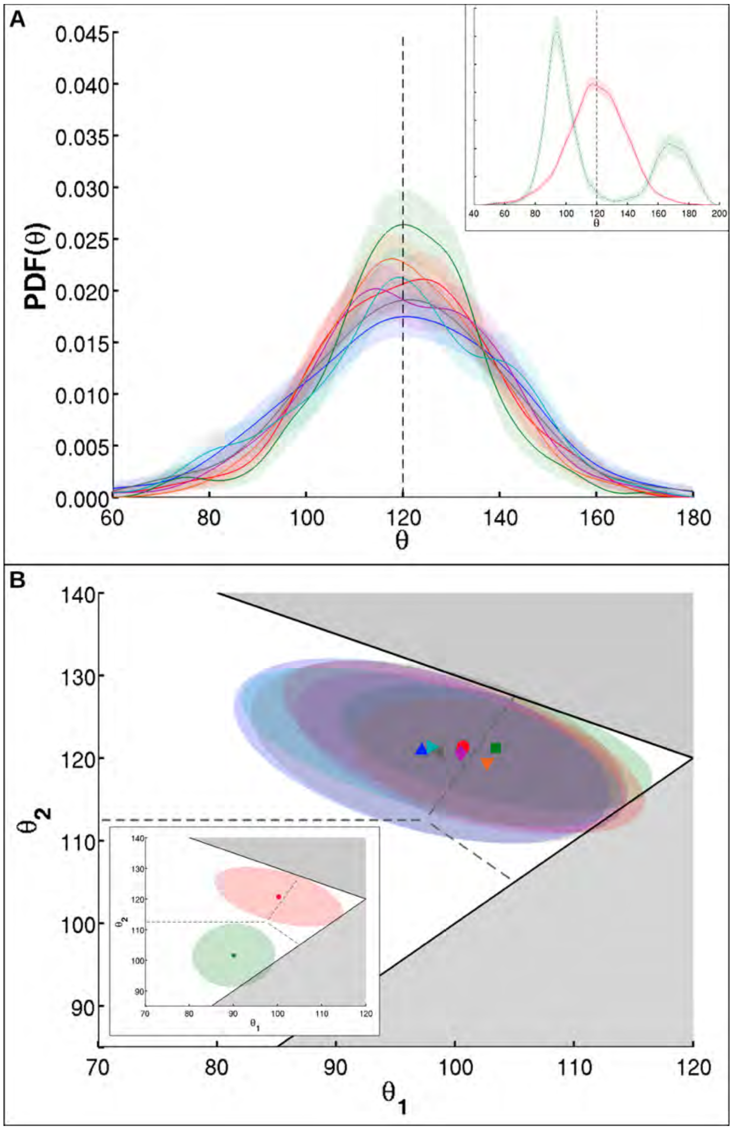

Supplementary Figure 15. Crack junctions exhibit a wide range of angular configurations. The probability density function (PDF) of vertex angles at crack junctions (**A**) exhibits a central, non-skewed peak close to 120º but this peak is substantially wider (~70% of the angles between 100º and 140º) than in trough junctions (Supplementary Fig 14). Moreover, the 2D angular scatter plots of crack junctions (**B,** and see Materials and Methods) show a marked spreading (average σ_θ = 14.1) between 'triple-120°' and '90°-135°-135°' profiles. These angular characteristics contrast starkly with those observed in free, planar cracking systems, such as, *e.g.*, montmorillonite desiccation. Inset of A: PDF of elephant skin (red, N=2997) and desiccated montmorillonite (green, N=1152) cracks. Inset of B: angular scatter plots of elephant skin (red, N=999) and montmorillonite (green, N=384) crack junctions. The angles were measured using skin photographs of living African elephants. Shaded regions represent 95% confidence intervals in (A) and 50% confidence regions in (B); see Supplementary Methods. Number of data points are given in Supplementary Table 3.

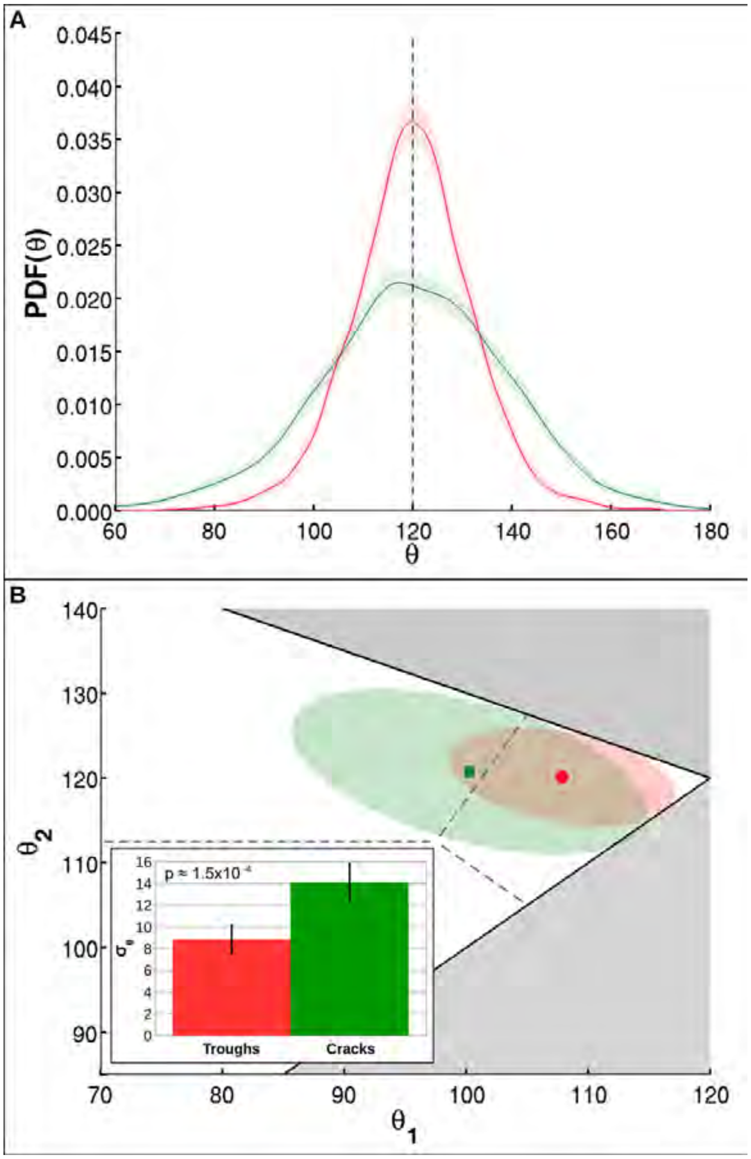

Supplementary Figure 16. The angular profiles of trough and crack junctions differ markedly. Although the probability density functions (PDFs) of both trough (red, N=3645) and crack (green, N=2997) junctions share a central peak close to 120° and a very small skewness, they exhibit a very clear difference in statistical dispersion (**A**). This is confirmed by the angular scatter plot (see Methods) of the two junction types (**B**): troughs (red, N=1215) and cracks (green, N=999); while troughs exhibit a clear tendency towards a 'triple-120°' profile with minor spreading (inset of B; average $\sigma_\theta = 8.8$, error bar = s.d. among 6 angular scatter plots of trough samples, Supplementary Table 2), the distribution of crack angles is significantly more dispersed (average $\sigma_\theta = 14.1$, error bar = s.d. among 7 angular scatter plots of observed crack samples, Supplementary Table 3, $p \approx 1.5\times10^{-4}$, Welch's *t*-test) varying roughly between the 'triple-120°' and the '90°-135°-135°' profiles. Shaded regions represent 95% confidence intervals in (A) and 50% confidence regions in (B); see Supplementary Methods.

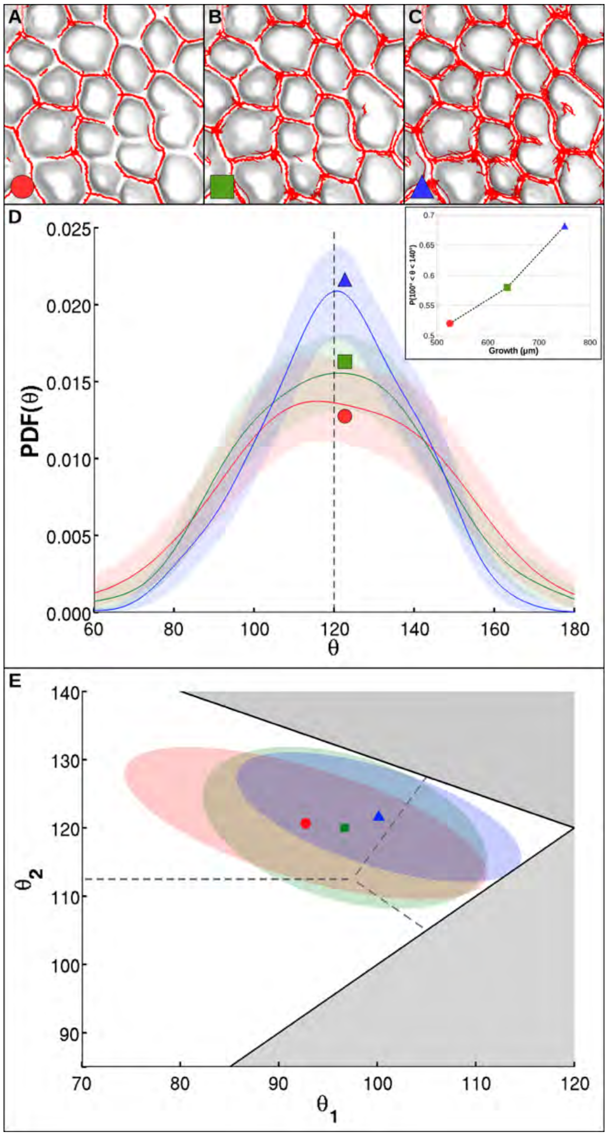

Supplementary Figure 17. The angular profiles of the simulated cracks undergo a process of maturation. (**A-C**) As the number of underlying layers of *stratum corneum* grows during the course of our numerical simulations, the pattern of cracks (red) evolves from a few junctions of narrow cracks (A) to one in which most troughs are cracked but little crack widening has taken place (B) to a stage in which cracks have widened considerably (C). (**D**) Statistically, the probability density function (PDF) of the vertex angles becomes progressively sharper around ~120°; red (N=81), green (N=153) and blue (N=186) curves refer to panels A, B, and C, respectively: the probability of measuring an angle between 100° and 140° rises from ~52% to ~68% (inset of D) in this example. (**E**) The angular scatter plot of the data (red, N=27; green, N=51; blue, N=62) shows that the pattern of simulated cracks evolves towards the 'triple-120°' profile, a trend usually accompanied by a mild decrease in angular spreading (here, from ~16.1 to ~13.4). Shaded regions represent 95% confidence intervals in (D) and 50% confidence regions in (E); see Supplementary Methods.

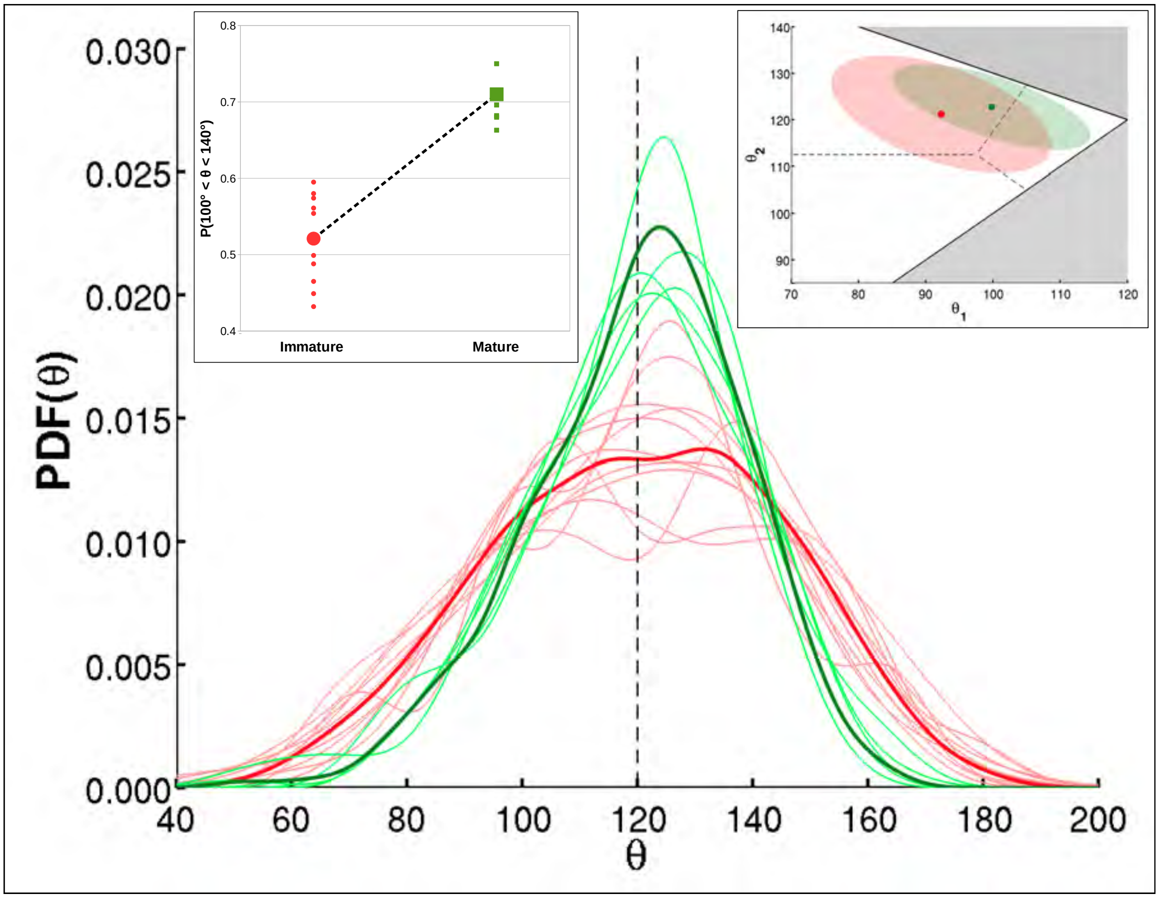

Supplementary Figure 18. The patterns of 'immature' and 'mature' simulated cracks differ markedly. Immature cracking patterns (red lines, thin indicates individual time points and thick their pooled average) have consistently shallower central peaks when compared to mature cracking networks (green lines, thin indicates individual samples and thick their pooled average). Left inset: the probability of finding an angle between 100° and 140° increases from ~52% to ~71% (small symbols indicate individual samples and large ones the values for the pooled distributions). Right inset: maturation causes a slight shift towards 'triple-120°' junctions, as evidenced by the angular scatter plot of the pooled results; red (N=531) and green (N=322) denote immature and mature patterns, respectively. Shaded regions represent 50% confidence regions (see Supplementary Methods).

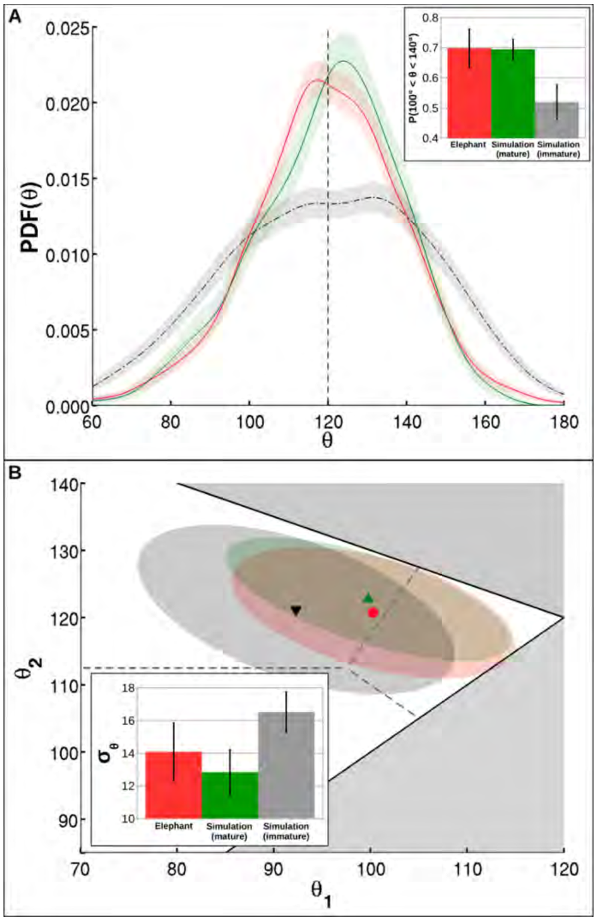

Supplementary Figure 19. Crack maturation drives the simulation system towards an angular distribution compatible with that observed in real skin cracks. (**A**) The probability density functions (PDF) of skin cracks (red line, N=2997) and mature simulated cracks (green line, N=966) show a very similar central peak sharpness. Contrastingly, the PDF of immature cracks (black dot-dashed line, N=1593) has a much broader angular dispersion, with a shallower central peak. Inset of A: probability of finding an angle between 100° and 140°, mean (error bars = s.d.) among 7 PDFs of skin cracks (red, Supplementary Table 3), 5 PDFs of mature simulated cracks (green, average of 193 angles per PDF), and 11 PDFs of immature simulated cracks (grey, average of 145 angles per PDF). (**B**) The angular scatter plots of the data additionally shows that mature (green triangle = average among 322 data points) and skin cracks (red circle = average among 999) have similar dispersions (between 'triple-120°' and '90°-135°-135°' profiles) and spreading. Immature simulated cracks (black triangle = average among 531 data points), on the other hand, exhibit markedly different average angles, as well as deviating dispersion direction and magnitude. Inset of B: angular spreading, mean (error bars = s.d.) among 7 angular scatter plots of skin cracks (red, Supplementary Table 3), 5 angular scatter plots of mature simulated cracks (green, average of 64 vertices per plot), and 11 angular scatter plots of immature simulated cracks (grey, average of 48 vertices per plot). Shaded regions represent 95% confidence intervals in (A) and 50% confidence regions in (B), see Supplementary Methods.

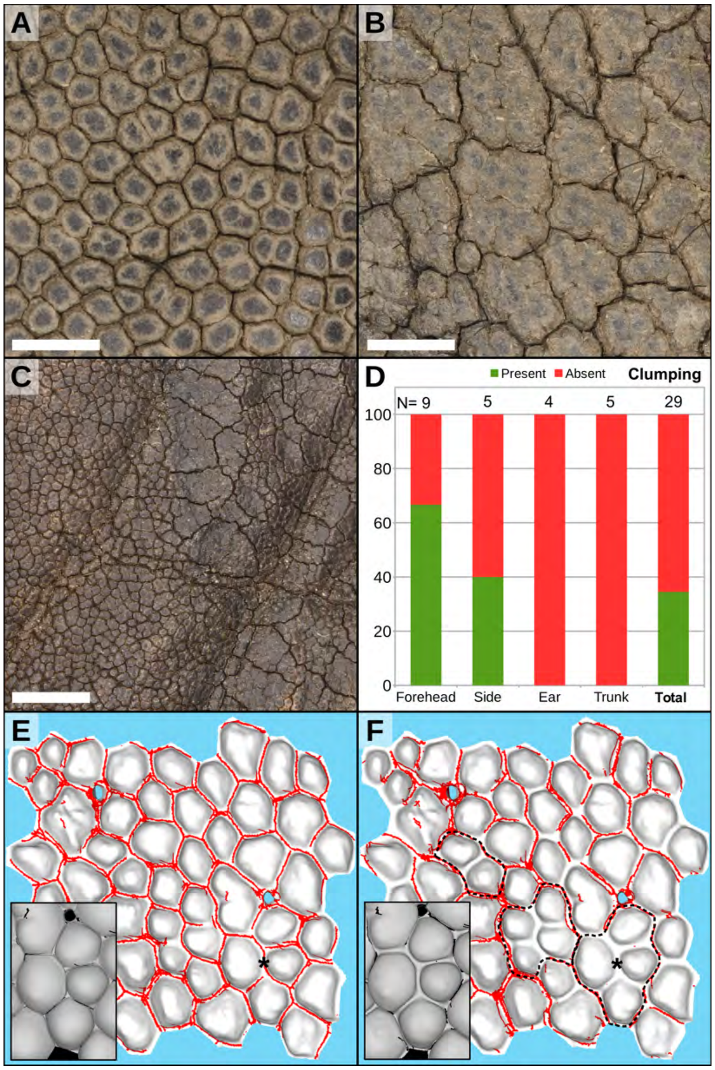

Supplementary Figure 20. Clumping of papillae. (**A**) In most cases, cracks appear on almost every trough, resulting in a skin pattern where papillae are largely individualized. (**B**) In some instances clumps containing a small number of papillae can form as the *stratum corneum* in-between them remains intact, giving a more 'patchy' appearance to the skin. (**C**) Sharp transitions between individualized and patchy patterns can sometimes be observed. (**D**) The clumping seems to occur mostly on the forehead and side of the animal, and never on more motile body parts such as the ear or the trunk. (**E,F**) Our model suggests that this phenomenon is related to the thickness of the outer *stratum corneum*, h, as no clumping is ever observed in simulations with $h/\langle d \rangle = 1/6$ (E), while it is sometimes seen when $h/\langle d \rangle = 2/6$ (dashed lines in F). $\langle d \rangle$ is the average trough spacing (~125-150μm). Results shown in (E) and (F) are both for $\varepsilon_c = 0.3$ and at a stage when ~3.5% of the mesh edges are cracked (red lines). Insets: close-up of the regions marked with an asterisk. The actual simulation mesh is shown, with the effects of *stratum corneum* growth included. Scale bar: (A) 5mm; (B) 7.5mm; (C) 5mm.

Supplementary Table 1: details of the specimens photographed for this study. We acquired close-up photographs from 16 animals, of which 10 were living or very recently deceased at the time of the acquisition. Pictures of the remaining 6 were obtained from fixed skin samples (labeled 'Fixed' and shaded in light gray). The group of photographed specimens includes both female and male animals with ages ranging from the fetal stages to adulthood. Several parts of the body were photographed, with special incidence on the forehead and side.

Animal	ID	Source	Age (years)	Sex	Body parts photographed
Heri	1	*Basel Zoo*<br>Basel (CH)	39	Female	Side
Malayka	2	*Basel Zoo*<br>Basel (CH)	44	Female	Forehead
N'dumé	3	*Réserve Africaine*<br>Sigean (FR)	32	Male	Forehead, Back, Buttocks, Shoulder
Makeba	4	*Zoo African Safari*<br>Plaisance-du-Touch (FR)	0<br>(newborn)	Female	Forehead, Side, Back
Chova	5	*Adventures with Elephants*<br>Bela Bela (RSA)	18	Male	Forehead, Side, Ear, Trunk, Back,
Chishuru	6	*Adventures with Elephants*<br>Bela Bela (RSA)	16	Male	Forehead, Side, Trunk
Shan	7	*Adventures with Elephants*<br>Bela Bela (RSA)	15	Female	Forehead, Side, Ear, Knee
Nuanedi	8	*Adventures with Elephants*<br>Bela Bela (RSA)	13	Female	Forehead, Back, Side, Trunk
Mussina	9	*Adventures with Elephants*<br>Bela Bela (RSA)	12	Female	Forehead, Side
Bela	10	*Adventures with Elephants*<br>Bela Bela (RSA)	0<br>(~20 days old)	Female	Forehead, Side
Fixed A	11	*University of the Witwatersrand*<br>Johannesburg (RSA)	25-30	Male	Forehead
Fixed B	12	*University of the Witwatersrand*<br>Johannesburg (RSA)	0<br>(~3 days old)	Unknown	Ear, Trunk
Fixed C	13	*University of Pretoria*<br>Pretoria (RSA)	~1	Unknown	Forehead, Ear, Trunk
Fixed D	14	*University of Pretoria*<br>Pretoria (RSA)	~1	Unknown	Forehead, Side, Ear, Trunk, Knee
Fixed E	15	*University of Pretoria*<br>Pretoria (RSA)	Fetus (<12 months gestation)	Unknown	Whole body
Fixed F	16	*University of Pretoria*<br>Pretoria (RSA)	Fetus (<12 months gestation)	Unknown	Whole body

CH – Switzerland; **FR** – France; **RSA** – Republic of South Africa.

Supplementary Table 2: samples used for trough junction angular statistics.

Animal ID	Suppl. Table 4 ID	Body part	Image acquisition	# of vertices	# of angles
14	T1	Ear	Micrograph	183	549
13	T2	Forehead (close to the trunk)	Micrograph	261	783
13	T3	Forehead (close to the ear)	Micrograph	425	1275
3	T4	Back	Micrograph	148	444
3	T5	Buttocks	Micrograph	128	384
12	T6	Ear	Simulation mesh screenshot	70	210

Supplementary Table 3: samples used for crack junction angular statistics.

Animal ID	Suppl. Table 4 ID	Body part	Image acquisition	# of vertices	# of angles
6	C1	Forehead	Photograph	152	456
6	C2	Forehead	Photograph	125	375
9	C3	Side	Photograph	128	384
8	C4	Forehead	Photograph	175	525
8	C5	Forehead	Photograph	141	423
8	C6	Side	Photograph	108	324
8	C7	Side	Photograph	170	510

Supplementary Table 4: summary of the angular statistics for trough and crack junctions. See 'Materials and Methods' for details. Trough statistics for the mesh used in the simulations are shaded in red.

ID	θ_1	θ_2	θ_3	σ_θ (1-sigma)	P(100° < θ < 140°)
T1	108.1	119.9	132.0	8.9	89.9%
T2	105.0	120.6	134.4	11.0	82.4%
T3	108.8	120.1	131.1	8.3	92.9%
T4	108.9	119.7	131.4	7.6	92.5%
T5	109.0	119.9	131.1	8.4	92.0%
T6	107.7	121.6	130.7	8.0	91.3%
C1	100.7	121.4	137.9	14.4	72.3%
C2	103.4	121.2	135.4	12.0	79.9%
C3	97.2	120.9	141.9	16.1	61.7%
C4	102.7	119.5	137.8	11.8	74.3%
C5	100.5	120.6	138.9	13.9	70.1%
C6	98.0	121.4	140.6	14.3	64.4%
C7	98.7	120.7	140.6	16.2	65.7%
Troughs (total)	107.9	120.2	131.9	9.1	90.5%
Cracks (total)	100.3	120.7	139.0	14.3	71.0%

Supplementary References

1. M. Ostoja-Starzewski, *Applied Mechanics Reviews* **55**, 35-60 (2002).
2. H. L. Chen, E. Q. Lin, Y. M. Liu, *Int J Solids Struct* **51**, 1819-1833 (2014).
3. E. Q. Lin, H. L. Chen, Y. M. Liu, *Finite Elem Anal Des* **93**, 1-11 (2015).
4. N. P. Mitchell, V. Koning, V. Vitelli, W. T. Irvine, *Nat Mater* **16**, 89-93 (2017).
5. Y. Kantor, D. R. Nelson, *Phys Rev Lett* **58**, 2774-2777 (1987).
6. H. S. Seung, D. R. Nelson, *Phys Rev A* **38**, 1005-1018 (1988).
7. P. G. Ciarlet, *Mathematical Elasticity, Volume III: Theory of Shells*. J. L. Lions, G. Papanicolaou, H. Fujita, H. B. Keller, Eds., (Elsevier, 2000).
8. N. Stoop, R. Lagrange, D. Terwagne, P. M. Reis, J. Dunkel, *Nat Mater* **14**, 337-342 (2015).
9. C. Ericson, *Real-Time Collision Detection*. (Taylor & Francis, 2004).
10. A. E. Shyer, T. Tallinen, N. L. Nerurkar, Z. Y. Wei, E. S. Gil, D. L. Kaplan, C. J. Tabin, L. Mahadevan, *Science* **342**, 212-218 (2013).
11. T. Tallinen, J. Y. Chung, F. Rousseau, N. Girard, J. Lefevre, L. Mahadevan, *Nat Phys* **12**, 588-593 (2016).
12. E. Cerda, L. Mahadevan, *Phys Rev Lett* **90**, (2003).
13. F. Brau, P. Damman, H. Diamant, T. A. Witten, *Soft Matter* **9**, 8177-8186 (2013).
14. L. D. Landau, E. M. Lifshitz, A. M. Kosevich, L. P. Pitaevskiĭ, *Theory of Elasticity*. (Butterworth-Heinemann, 1986).
15. N. Bowden, S. Brittain, A. G. Evans, J. W. Hutchinson, G. M. Whitesides, *Nature* **393**, 146-149 (1998).
16. K. Levi, R. J. Weber, J. Q. Do, R. H. Dauskardt, *Int J Cosmet Sci* **32**, 276-293 (2010).
17. K. Levi, Stanford University, (2009).
18. M. Geerligs, "A literature review of the mechanical behavior of the stratum corneum, the living epidermis and the subcutaneous fat tissue," *No. Philips Research Europe* (2006).
19. Y. S. Papir, K. H. Hsu, R. H. Wildnauer, *Biochim Biophys Acta* **399**, 170-180 (1975).
20. P. J. Caspers, G. W. Lucassen, H. A. Bruining, G. J. Puppels, *J Raman Spectrosc* **31**, 813-818 (2000).
21. P. J. Caspers, G. W. Lucassen, E. A. Carter, H. A. Bruining, G. J. Puppels, *Journal of Investigative Dermatology* **116**, 434-442 (2001).
22. J. M. Crowther, A. Sieg, P. Blenkiron, C. Marcott, P. J. Matts, R. Kaczvinsky, A. V. Rawlings, *Brit J Dermatol* **159**, 567-577 (2008).
23. S. J. Sheather, *Stat Sci* **19**, 588-597 (2004).
24. S. J. Sheather, M. C. Jones, *J Roy Stat Soc B Met* **53**, 683-690 (1991).
25. H. B. Lillywhite, B. R. Stein, *J Zool* **211**, 727-734 (1987).
26. R. C. Dunkin, D. Wilson, N. Way, K. Johnson, T. M. Williams, *Journal of Experimental Biology* **216**, 2939-2952 (2013).
27. L. Goehring, *Philos Trans A Math Phys Eng Sci* **371**, 20120353 (2013).
28. P. Nandakishore, L. Goehring, *Soft Matter* **12**, 2253-2263 (2016).
29. L. Goehring, *Phys Rev E Stat Nonlin Soft Matter Phys* **80**, 036116 (2009).
30. L. Goehring, R. Conroy, A. Akhter, W. J. Clegg, A. F. Routh, *Soft Matter* **6**, 3562-3567 (2010).
31. P. Fleckman, S. Brumbaugh, *Experimental dermatology* **11**, 327-336 (2002).
32. P. M. Elias, K. R. Feingold, *Skin Barrier*. (CRC Press, 2005).
33. M. Schmuth, R. Gruber, P. M. Elias, M. L. Williams, *Adv Dermatol* **23**, 231-256 (2007).
34. R. I. Spearman, *Zoologica Africana* **5**, 327-338 (1970).
