# Supplementary material for: Locally-curved geometry generates bending cracks in the African elephant skin
Source: Nat Commun. 2018 Oct 2;9:3865. doi: 10.1038/s41467-018-06257-3 (PMC6168576; doi:10.1038/s41467-018-06257-3)
Supplement: Supplementary file 3 — Description of Additional Supplementary Files [file 41467_2018_6257_MOESM3_ESM.pdf]

Description of Additional Supplementary Files

File Name: Supplementary Movie 1
Description: *In vivo* wetting of African bush elephant skin. The application (using a syringe) of 10mL of water at a localized point on the side of the animal demonstrates the remarkable water retention and spreading potential of its skin. A band of paper of size 15cm is positioned above the application point. Names and ID (cf. Supplementary Table 1) are shown for the three animals used for this experiment.

File Name: Supplementary Movie 2
Description: Close-up of an *in vivo* wetting of African bush elephant skin. When water is applied at a localized point on the side of the animal, it initially flows quickly (up to ~2-3 cm/s) and against gravity (probably due to capillary action), resulting in a rapid and thorough wetting of the nearby skin areas. The sides of squares on the paper on the left of the application point are 0.5 cm.

File Name: Supplementary Movie 3
Description: Mechanical removal of the *stratum corneum* from a fixed sample (I). When the *stratum corneum* is mechanically extracted from the elephant's skin, it tends to come off in patches delimited by cracks, suggesting that the layer is more fragile in these regions (probably as a consequence of being thinner there, see supplementary Discussion).

File Name: Supplementary Movie 4
Description: Mechanical removal of the *stratum corneum* from a fixed sample (II). In some cases, a patch of *stratum corneum* containing cracks can be detached at once, indicating that the layer is continuous (albeit feebly, see Supplementary Movie 3) even in cracked regions. This is consistent with our light and electron microscopy observations (see supplementary Discussion).

File Name: Supplementary Movie 5
Description: Numerical simulation of the inner *stratum corneum* growth process. As more *stratum corneum* sheets are formed at the *stratum basale*, the outer *stratum corneum* (left) is pushed outwards, develops stress and eventually cracks (right). In this example, the thickness of the inner *stratum corneum* is increased by ~2µm per step (*i.e.*, $\varepsilon \approx 2\mu m$). The thickness of the outer *stratum corneum* (h) is ~20µm, and the elongation at break (ε_c) is 0.3.
